# Mechanism of ribonucleic acid-binding protein ILF2 in promoting diabetic foot ulcer wound healing via regulating the nucleophosmin 1/NF-κB axis

**DOI:** 10.1093/burnst/tkag021

**Published:** 2026-03-17

**Authors:** Hua Ji, Ying Tang, Chenfan Zhang, Yinguang Jia, Murong Xu, Xiaotong Zhao, Mingwei Chen

**Affiliations:** Department of Endocrinology, The First Affiliated Hospital of Anhui Medical University, Shushan District, 218 Jixi Road, Hefei, Anhui, 230022, China; Institute of Endocrinology and Metabolism, Anhui Medical University, No. 81 Meishan Road, Shushan District, Hefei, Anhui, 230032, China; Department of Endocrinology, The First Affiliated Hospital of Anhui Medical University, Shushan District, 218 Jixi Road, Hefei, Anhui, 230022, China; Institute of Endocrinology and Metabolism, Anhui Medical University, No. 81 Meishan Road, Shushan District, Hefei, Anhui, 230032, China; Department of Endocrinology, The First Affiliated Hospital of Anhui Medical University, Shushan District, 218 Jixi Road, Hefei, Anhui, 230022, China; Institute of Endocrinology and Metabolism, Anhui Medical University, No. 81 Meishan Road, Shushan District, Hefei, Anhui, 230032, China; Department of Endocrinology, The First Affiliated Hospital of Anhui Medical University, Shushan District, 218 Jixi Road, Hefei, Anhui, 230022, China; Institute of Endocrinology and Metabolism, Anhui Medical University, No. 81 Meishan Road, Shushan District, Hefei, Anhui, 230032, China; Department of Endocrinology, The First Affiliated Hospital of Anhui Medical University, Shushan District, 218 Jixi Road, Hefei, Anhui, 230022, China; Institute of Endocrinology and Metabolism, Anhui Medical University, No. 81 Meishan Road, Shushan District, Hefei, Anhui, 230032, China; Department of Endocrinology, The First Affiliated Hospital of Anhui Medical University, Shushan District, 218 Jixi Road, Hefei, Anhui, 230022, China; Institute of Endocrinology and Metabolism, Anhui Medical University, No. 81 Meishan Road, Shushan District, Hefei, Anhui, 230032, China; Department of Endocrinology, The First Affiliated Hospital of Anhui Medical University, Shushan District, 218 Jixi Road, Hefei, Anhui, 230022, China; Institute of Endocrinology and Metabolism, Anhui Medical University, No. 81 Meishan Road, Shushan District, Hefei, Anhui, 230032, China

**Keywords:** Diabetic foot ulcer, Interleukin enhancer-binding factor 2, RNA binding protein, Senescence-associated secretory phenotype, NF-κB axis, Nucleophosmin, Wound healing, Fibroblast, Migration, Senescence

## Abstract

**Background:**

Diabetic foot ulcer (DFU) is a severe diabetic complication characterized by impaired healing, often involving fibroblast senescence and the senescence-associated secretory phenotype (SASP). The role of ribonucleic acid (RNA)-binding proteins (RBPs) in this process remains undefined. This study investigates the function and mechanism of the RBP interleukin enhancer-binding factor 2 (ILF2) in DFU pathogenesis.

**Methods:**

Differentially expressed RBPs were identified via bioinformatics analysis of public single-cell and bulk transcriptomic datasets. ILF2 downregulation was subsequently validated in clinical DFU samples and diabetic mouse models. Functional assays in high-glucose (HG)-treated fibroblasts evaluated proliferation, migration, and SASP. Mechanistically, RNA sequencing, RNA-binding protein immunoprecipitation, and RNA pull-down assays identified downstream targets, while co-IP and rescue experiments verified the NPM1/NF-κB axis. Finally, a diabetic mouse model was used to study the effects of ILF2 overexpression/knockdown and NPM1 knockdown on wound healing.

**Results:**

Bioinformatics analysis identified ILF2 as significantly downregulated in DFU. This reduction was consistently validated in DFU patient tissues, diabetic mouse wounds, and HG-treated fibroblasts. Functionally, ILF2 overexpression promoted fibroblast proliferation and migration while suppressing SASP, whereas knockdown exacerbated senescence. Mechanistically, ILF2 directly bound to nucleophosmin (NPM1) mRNA to promote its degradation. ILF2 deficiency led to aberrant NPM1 accumulation, enhancing the NPM1–phospho-p65 interaction and NF-κB pathway activation. Rescue experiments confirmed that NPM1 knockdown reversed ILF2 deficiency-induced cellular dysfunction. Crucially, these findings were validated in primary fibroblasts isolated from DFU patients. *In vivo*, ILF2 overexpression accelerated wound healing, while knockdown delayed the process. Furthermore, NPM1 knockdown effectively ameliorated the impaired healing phenotype and reduced SASP levels.

**Conclusions:**

This study elucidates a novel ILF2–NPM1–NF-κB regulatory axis. ILF2 acts as a critical suppressor of inflammatory senescence by destabilizing NPM1 mRNA, highlighting its potential as a therapeutic target for DFU treatment.

## Graphical Abstract

**Graphical Abstract:**
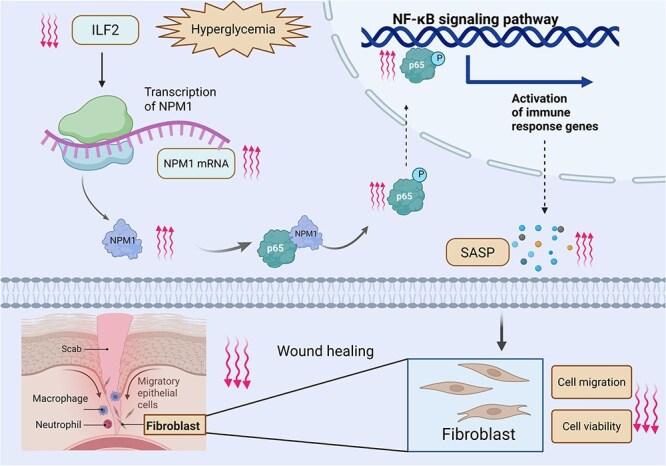

## Highlights

- The ribonucleic acid-binding protein interleukin enhancer-binding factor 2 (ILF2) is significantly downregulated in fibroblasts from diabetic foot ulcer tissues.
- ILF2 promotes the degradation of nucleophosmin 1 (NPM1) mRNA by directly binding to it, thereby inhibiting NF-κB signaling and the senescence-associated secretory phenotype.
- Restoring ILF2 expression accelerates diabetic wound healing by targeting the NPM1/NF-κB axis to alleviate inflammatory senescence.

## Background

Diabetic foot ulcer (DFU) represent a common and highly morbid consequence of long-standing, poorly controlled diabetes, characterized by complex underlying pathophysiology. Among the estimated 537 million individuals with diabetes globally [1], 19%–34% will develop DFU during their lifetime. Furthermore, 17% of patients with DFU-related complications undergo lower limb amputations—minor (below ankle) or major [2]. Notably, the severity of DFU leads to a 10% mortality rate within one year after initial diagnosis [3]. Therefore, exploring the molecular mechanisms underlying impaired skin wound healing in diabetes and identifying new key therapeutic targets is of significant clinical importance.

Skin wound healing is a complex process involving several stages, including coagulation, inflammation, proliferation, and remodeling [4]. This process requires the coordinated actions of endothelial cells, fibroblasts, immune cells, growth factors, and the extracellular matrix [5]. Fibroblasts play a crucial role in the healing process by proliferating, migrating, and secreting collagen and fibronectin to form granulation tissue, and by differentiating into myofibroblasts to promote wound contraction and re-epithelialization [6]. However, in the diabetic microenvironment, high glucose (HG) levels and advanced glycation end products induce oxidative stress, inflammatory responses, mitochondrial dysfunction, and cellular senescence [6, 7]. Cellular senescence [8] is a state of terminal growth arrest associated with the upregulation of different cell cycle inhibitors, structural and metabolic alterations, chronic deoxyribonucleic acid (DNA) damage responses, and a hypersecretory state known as the senescence-associated secretory phenotype (SASP). The SASP is the major mediator of the paracrine effects of senescent cells in their tissue microenvironment and of various local and systemic biological functions. Recent advances elucidate the temporal duality of the SASP in skin biology: while it orchestrates essential regeneration and repair in acute injuries such as burns, its chronic persistence drives pathological fibrosis and impairs tissue homeostasis， [8–10]. Consistent with this pathological role, Shang *et al*. [11] discovered that diabetic mice accumulate a significant number of senescent cells, predominantly fibroblasts, in their skin and wound tissues. Further studies have indicated that such senescent fibroblasts impair healing in chronic wounds, such as DFU, by secreting persistent SASP that disrupts tissue homeostasis [12–15]. Guided by the major SASP factors identified by Wang *et al*. [8], we selected representative targets to comprehensively evaluate SASP expression in our fibroblasts, covering distinct functional categories: IL-1β as a primary upstream regulator; IL-6 and IL-8 as major mediators of paracrine senescence and immune response; and MMP1 and MMP3 as critical proteases associated with extracellular matrix degradation and skin aging.

Ribonucleic acid (RNA)-binding proteins (RBPs) [16] are key regulators of RNA stability, splicing, transport, and translation, influencing critical biological processes like immune response, cellular stress, metabolism, and tumor igenesis. Emerging evidence now underscores their specific contributions to skin repair in both burn injuries and chronic wounds. In acute burn wounds, certain RBPs accelerate healing by modulating angiogenesis and tissue remodeling, for example, LIN28B [17] promotes repair after thermal injury by upregulating VEGFA and miR-21 to enhance neovascularization. Furthermore, lincRNA TINCR [18] binds to SND1 to upregulate TGF-β1, thereby promoting skin fibroblast proliferation and exacerbating post-burn inflammation. In the context of chronic, non-healing wounds such as DFU, RBPs have been identified as critical regulators of multiple repair processes. For instance, the RBP Acheron [19] promotes endothelial cell proliferation following injury. TATA-box binding protein associated factor 15 (TAF15) [20] binds to and stabilizes Nrf2 mRNA, thereby activating the Nrf2 signaling pathway to facilitate DFU healing. Furthermore, YTH domain-containing protein 1 (YTHDC1) [21] regulates the mRNA stability of SQSTM1 to induce keratinocyte autophagy, thus promoting diabetic wound repair. Given the critical roles of inflammation and cellular proliferation in these reparative processes, interleukin enhancer-binding factor 2 (ILF2) [22] is of particular interest. ILF2 is a multifunctional protein pivotal to cell growth, inflammation, DNA damage repair, and division. Notably, ILF2 has also been shown to promote cancer cell proliferation and migration [23, 24], while inhibiting NLRP3 inflammasome activation to modulate macrophage inflammatory responses, particularly in diabetic environments [25, 26]. However, its role in skin fibroblasts remains unknown. Considering that fibroblasts are the primary source of pathological SASP in diabetic wounds, coupled with ILF2’s functions in genomic stability and inflammation, we hypothesize that ILF2 modulates fibroblast senescence. Elucidating this mechanism could uncover novel therapeutic targets to reverse senescence and improve DFU healing.

In this study, we performed clustering analysis of fibroblasts using public single-cell RNA sequencing (RNA-seq) data from DFU patients and identified the aberrant downregulation of ILF2. Consistent reductions were validated in the wounds of both DFU patients and diabetic mice. Through clinical and cellular experiments, we demonstrated that ILF2 promotes fibroblast proliferation and migration while suppressing the SASP. To elucidate the underlying mechanism, we identified nucleophosmin (NPM1) as a direct downstream target of ILF2. We further revealed that ILF2 binds directly to NPM1 mRNA to induce its degradation, thereby regulating NPM1 expression at the post-transcriptional level. Consequently, ILF2 downregulation under HG conditions led to NPM1 accumulation, which subsequently activated the NF-κB pathway via interaction with phospho-p65 (p65), driving SASP aggravation. Finally, we validated these findings in a diabetic wound mouse model using adeno-associated virus (AAV)-mediated ILF2 knockdown and overexpression, as well as AAV-mediated NPM1 knockdown. This study provides new insights into the pathogenesis of DFU and may offer potential therapeutic targets for future treatments.

## Methods

### Human wound samples

All samples were obtained from hospitalized patients at Anhui Medical University First Affiliated Hospital between October 2024 and June 2025, including wound margin tissue samples from 8 DFU patients admitted to the Department of Endocrinology and 8 non-diabetic trauma patients (defined as the control group, NC) admitted to the Department of Burn Surgery. Inclusion criteria for the DFU group were: age 18–80 years, foot ulcer duration >4 weeks, ulcer area 2–20 cm^2^, and Ankle–Brachial Index 0.9–1.3. All clinical experiments were approved by the Ethics Committee of Anhui Medical University First Affiliated Hospital (Approval No. PJ-2025-02-34), the clinical trial was registered under ChiCTR2400088416, and written informed consent was obtained from all participants. The clinical information of patients was shown in Table S1.

#### Cell culture

Human skin fibroblasts (BJ cell line, GNHu49) were obtained from the Cell Bank of the Chinese Academy of Sciences (Shanghai, China). Cells were maintained in HG Dulbecco’s Modified Eagle Medium (DMEM; Gibco, USA) supplemented with 10% fetal bovine serum (FBS), 100 IU/ml penicillin, and 100 μg/ml streptomycin. Cultures were incubated at 37°C in a humidified atmosphere containing 5% CO₂. For HG stimulation experiments, cells were cultured in medium supplemented with D-glucose (Sigma-Aldrich, USA) to a final concentration of 50 mM for 7 days. In addition, to confirm whether the results were influenced by osmotic pressure, a supplementary experiment was conducted comparing the high mannitol (HM) group (5.5 mM D-glucose +44.5 mM mannitol) with HG group.

#### Cell transfection

Detailed transfection procedures are described below, taking a 24-well plate format as an example. One day before transfection, cells were seeded to achieve a confluence of approximately 50%–60% at the time of transfection, a density optimized through multiple preliminary experiments to prepare the transfection complex, 30 pmoles of siRNA (GeneAdv Co. Ltd, China) were diluted in 100 μl of serum-free medium (Opti-MEM). Subsequently, 2–4 μl of the transfection reagent (Yeasen Biotechnology, 40806ES03, Shanghai) was added to the diluted siRNA. The mixture was vortexed thoroughly for 10 s and incubated at room temperature for 10–15 min to form the siRNA-transfection reagent complex (incubation time was strictly controlled not to exceed 30 min). During the incubation period, the original culture medium was aspirated and replaced with 500 μl of fresh, pre-warmed complete medium. The 100 μl complex was then added directly to each well, resulting in a final volume of 600 μl and a final siRNA concentration of 50 nM. The plate was gently shaken to ensure even distribution. For co-transfection (rescue) experiments, the total amount of siRNA was kept consistent with that used in individual transfections to control for transfection efficiency and cytotoxicity. Specifically, while 30 pmoles of a single siRNA were used for individual transfections, the co-transfection groups received a mixture of two different siRNAs (e.g. 15 pmoles of ILF2 siRNA +15 pmoles of NPM1 siRNA), maintaining the total siRNA quantity at 30 pmoles. Following transfection, cells were incubated at 37°C in a 5% CO2 humidified incubator. The efficiency of gene knockdown was assessed at the mRNA level 48 h post-transfection and at the protein level 72 h post-transfection. The siRNA sequences used in this study are listed in Table S2. For ILF2 overexpression, an empty vector (oe-NC) was used as a negative control. The ILF2 overexpression construct containing the full-length encoding sequence (NM_026374.3) was transfected into fibroblasts. The detailed sequences for the overexpression constructs are provided in Table S2.

### Reverse transcription quantitative polymerase chain reaction

Total RNA was extracted from tissues or cells using TRIzol reagent, followed by reverse transcription into cDNA. Reverse transcription quantitative polymerase chain reaction (RT-qPCR) was performed using SYBR Green, with primers synthesized by Sangon Biotech (Shanghai, China) (sequences listed in Table S3). Gene expression levels were calculated using the 2^− ΔΔCt^ method.

#### Western blot

Total protein was extracted from human samples, BJ cell, and mouse skin wound tissues using the Total Protein Extraction kit (Solarbio, Beijing, China). The protein concentration was determined using the bicinchoninic acid protein assay (Keygene Biotech, Nanjing, China). After separation on a 10% sodium dodecyl sulfate-polyacrylamide gel electrophoresis gel, the protein was transferred to polyvinylidene fluoride membranes (Millipore, USA). The membranes were blocked with non-fat powdered milk (Beyotime, Shanghai, China) in Tris-buffered saline with Tween-20 and then incubated with primary and secondary antibodies (Table S4). Finally, the protein blots were detected using enhanced chemiluminescence reagents (Vazyme, Nanjing, China) and recorded with the Tanon Chemi-Image System.

#### Cell counting kit-8 assay

BJ were harvested, resuspended, and seeded at a density of 1 × 10^3 cells/well in a 96-well plate. Ten μl of cell counting kit-8 solution (Biosharp Life Sciences) was added to each well at the indicated time. After a 2-h incubation, the absorbance (A) at 450 nm was measured using a microplate reader. A standard curve was generated to calculate the cell proliferation rate following the manufacturer’s instructions.

#### Wound healing assay

Logarithmic phase cells were seeded at 1 × 10^6^ cells/well into a 6-well plate, with three replicates per group. Once cells reached 90%–100% confluence, a 200-μl pipette tip was used to create a vertical scratch. After removing the detached cells with PBS (three washes), serum-free DMEM was added, and the plate was incubated at 37°C with 5% CO2. Images were captured at 0 and 48 h using an optical microscope. The wound healing rate was calculated using ImageJ (version 1.8.0) and the formula: **wound healing rate (%) = [(0 h scratch area - 48 h scratch area)/0 h scratch area] × 100.**

#### 5-Ethynyl-2′-deoxyuridine assay

5-Ethynyl-2′-deoxyuridine (EdU) assay was undertaken with BeyoClick™ EdU Cell Proliferation Kit with Alexa Fluor 594 (Beyotime, Shanghai, China). After washing in PBS, EdU solution was used to incubate cells for 2 h. Cell nuclei were then stained by DAPI solution. After washing, samples were observed with an inverted microscope (Olympus).

#### Immunofluorescence

Cell-climbing slides were fixed with 4% paraformaldehyde and permeabilized with 0.5% Triton X-100 at room temperature for 20 min. For paraffin sections, after dewaxing and antigen retrieval, the sections were blocked with 3% BSA for 30 min. Following removal of the blocking solution, the sections were incubated with the primary antibody against ILF2 (1:100) at 4°C overnight in a humidified chamber. The next day, HRP-conjugated secondary antibody was added and incubated at room temperature for 50 min. After washing with PBS, the sections were gently drained and incubated with TSA dye (ZENBIOSCIENCE, China) for 10 min at room temperature in the dark. Subsequently, the sections were incubated with the primary antibody against NPM1 (1:100) at 4°C overnight in a humidified chamber. After PBS washing the following day, the appropriate fluorescent secondary antibody was added and incubated for 50 min at room temperature in the dark. After PBS washing, the sections were mounted with an antifade mounting medium containing DAPI and observed under a fluorescence microscope. Images were captured and saved for further analysis.

### Bioinformatics analysis

The scRNA-seq dataset of GSE165816 was retrieved from the Gene Expression Omnibus (GEO) database (http://www.ncbi.nlm.nih.gov/geo/). For this analysis, data from seven healed-DFU and four unhealed-DFU samples were selected (version 4.3.2).The baseline characteristics of the patients are listed in Table S5. The samples were converted to Seurat objects using the ‘Create Seurat Object’ function, filtering for cells with 200–8000 features and genes expressed in at least three cells. Mitochondrial gene percentages were calculated using the ‘Percentage Feature Set’ function, and cells with a mitochondrial gene content exceeding 20% were excluded. Data integration was performed using the ‘FindIntegrationAnchors’ function, followed by selection of the top 3000 highly variable genes using the ‘SelectIntegrationFeatures’ function. The data were then scaled, and principal component analysis (PCA) was performed. Cell clustering was conducted using the ‘FindNeighbors’ and ‘FindClusters’ functions, with a resolution of 0.3. cell type annotations were predicted using the SingleR package. Differentially expressed genes (DEGs) for each cell subgroup were identified with the ‘FindAllMarkers’ function (min.pct = 0.25, logfc.threshold = 1, *P* < 0.05). Cell clustering and annotation were further refined based on known cell-type markers obtained from the literature [27]. UMAP and Stream Graphs were used to visualize clustered cells extracted from DFU and healthy control tissues. Additionally, the GSE199939 bulk RNA-seq dataset was also downloaded from the GEO database, containing 10 DFU samples and 11 normal samples, and DEGs were visualized using violin plots. The dataset includes 10 DFU patients (61.9 ± 7.6 years, 5 males and 5 females) and 11 healthy subjects (60.91 ± 8.8 years, 5 males and 6 females).

### RNA sequencing

The control group was HG-ctrl fibroblasts, and the experimental group was HG-si ILF2 fibroblasts. Total RNA was extracted using TRIzol reagent, and RNA purity and concentration were assessed using the NanoDrop 2000 spectrophotometer (Thermo Scientific, USA). RNA integrity was evaluated using the Agilent 2100 Bioanalyzer (Agilent Technologies, Santa Clara, CA, USA). Library construction was performed using the VAHTS Universal V6 RNA-seq Library Prep Kit according to the manufacturer’s instructions. Transcriptome sequencing and subsequent data analysis were conducted by OE Biotech Co., Ltd (Shanghai, China). Differential gene expression analysis was performed using DESeq2, with genes having a q-value <0.05 and fold change >0.5 considered significantly differentially expressed. Hierarchical clustering of DEGs was performed in R (version 3.2.0) to visualize the expression patterns across different groups and samples. Gene Ontology (GO), Kyoto Encyclopedia of Genes and Genomes (KEGG) pathway, Reactome, and WikiPathways enrichment analyses were performed based on the hypergeometric distribution algorithm to identify significantly enriched functional categories. Enrichment circular plots were generated in R to assess the concentration of genes in the defined list based on differential expression levels between the two sample groups.

### RNA-binding protein immunoprecipitation

RNA-binding protein immunoprecipitation (RIP) was conducted using the RIP kit from BersinBio (China) according to the manufacturer’s protocol. Cells were lysed with an appropriate volume of RIP lysis buffer, and RBPs were immunoprecipitated using an anti-ILF2 antibody or normal rabbit IgG. The co-precipitated RNA was purified and resuspended in RNasefree water. Target RNA enrichment was analyzed via RT-qPCR.

### RNA pull-down assays

RNA pull-down assays were performed using both sense and antisense NPM1 probes. Following the guidelines from BersinBio (China), the reagents were used to enrich proteins that bind to NPM1 mRNA. The enriched proteins were then analyzed by WB.

### RNA stability assay

RNA stability was assessed as described previously [28]. Fibroblasts were seeded in a 6-well plate and transfected when they reached ~70% confluence (ctrl; and oe-ILF2). After 48 h, actinomycin D (5 μg/ml) was added at different time points to inhibit RNA transcription. Cells were then collected at the designated time points, total RNA was extracted, and RNA degradation was evaluated by RT-qPCR.

### Co-immunoprecipitation assay

For co-immunoprecipitation assay (Co-IP) analysis, fibroblast cells were lysed with IP buffer (P0013F, Beyotime Biotechnology), followed by centrifugation at 12 000 × g for 30 min at 4°C. Protein A/G magnetic beads (P2055, Beyotime Biotechnology) were incubated with the corresponding antibody (1:100 dilution) or normal IgG as a negative control at 4°C for 4 h to form antibody-coated magnetic beads. The supernatant was then incubated with the magnetic bead-antibody complex on a shaker at 4°C overnight. After incubation, the beads were separated from the cell lysate using a magnet and washed with wash buffer.

### Animal experiments

All animal experiments were approved and performed in accordance with the guidelines of the Animal Care and Use Committee of Anhui Medical University (Hefei, China; approval no. LLSC20201040). Male C57BL/6 mice (6 weeks old, 18–20 g) were purchased from GemPharmatech Co., Ltd, raised in controlled habitats and provided with water and food.

Diabetes was induced in mice by intraperitoneal injection of streptozotocin (STZ; Sigma-Aldrich, USA) dissolved in 0.1 M sodium citrate buffer (pH 4.5). Mice received daily injections of STZ at a dose of 50 mg/kg body weight for five consecutive days. Beginning on Day 12 after the first injection, fasting blood glucose levels were measured twice weekly from tail vein blood samples for 2 weeks. Mice were considered diabetic when random blood glucose levels were ≥ 250 mg/dL (16.7 mmol/L) on two consecutive measurements. Blinding was not applied in this study.

To compare the wound healing differences between diabetic and normal wounds, sterile skin punch biopsies were performed on the dorsal skin of both diabetic mice and control group mice to create full-thickness wounds. To investigate the expression patterns of ILF2, NPM1, and senescence markers during the wound healing process, mice were euthanized at predetermined time points, and wound tissues were harvested for subsequent analysis. This included hematoxylin and eosin (H&E) staining, MT staining, RT-qPCR, WB, IHC, and IF analysis.

To investigate the role of ILF2 and NPM1 *in vivo*, both AAV-mediated knockdown and overexpression systems were employed (Shanghai GenePharma, Ltd). For ILF2 knockdown, an AAV vector carrying a short hairpin RNA targeting ILF2 (AAV-shILF2) was constructed (shRNA sequence: 5′-CCGGCAGGTAGGATCATATAA-3′). For NPM1 knockdown, an AAV vector carrying a short hairpin RNA targeting NPM1 (AAV-shNPM1) was constructed (shRNA sequence: 5′-CTATGAAGGCAGTCCAATTAA-3′). An AAV vector expressing a non-targeting shRNA (AAV-shRNA NC, sequence: 5′-ACTACCGTTGTTATAGGTGGTG-3′) served as a negative control for the knockdown experiments. For ILF2 overexpression, an AAV vector (AAV-OE-ILF2) carrying the *Mus musculus* Ilf2 coding sequence (NM_026374.3) was generated. An empty AAV vector without an insert was used as the control. In all experiments, the dorsal hair of mice was shaved, and the respective viral vectors were injected subcutaneously into the dorsal skin at a dose of 1.0 × 10^11 vector genomes per mouse in 100 μl PBS.

Intradermal Injection. The intradermal injection was performed as follows: the specific operation steps were as follows: (i) skin preparation: the animals were shaved, and a depilatory cream was applied to ensure the skin was clearly visible. (ii) Marking and injection: the target injection area was first outlined with a black marker. The skin near the injection site was gently pinched, and a needle (attached to a 1 ml syringe) was inserted into the skin at a 15°–30° angle. (iii) Confirmation and Completion: Successful injection was confirmed by the appearance of a small, pale bleb (wheal) at the site, which appears whiter than the surrounding skin. After the injection, gentle pressure was applied to prevent backflow.

At the indicated time points, skin tissues were harvested, and ILF2 expression levels were evaluated by RT-qPCR to confirm the efficiency of knockdown or overexpression.

Mice were anesthetized by intraperitoneal injection of 1% pentobarbital (50 mg/kg) and then disinfected with 5% iodophor disinfectant. After drying, the skin was disinfected with 75% ethanol. Under anesthesia, two symmetrical full-thickness skin excisional wounds were created near the midline of the dorsal skin using a 6-mm biopsy punch. For the ILF2 knockdown experiments, mice were divided into the following groups: normal control (non-sh ILF2-ctrl), normal knockdown (non-sh ILF2), diabetic control (DM-sh ILF2-ctrl), and diabetic knockdown (DM-sh ILF2). For the NPM1 knockdown experiments, mice were divided into: normal control (non-sh NPM1-ctrl), normal knockdown (non-sh NPM1), diabetic control (DM-sh NPM1-ctrl), and diabetic knockdown (DM-sh NPM1). The overexpression group was divided into: normal control (non-oe ILF2-ctrl), normal overexpression (non-oe ILF2), diabetic control (DM-oe ILF2-ctrl), and diabetic overexpression (DM-oe ILF2).

The wound healing process was documented by photography. On Day 6, a 3-mm full-thickness skin biopsy was taken from the wound edge for subsequent analyses, including H&E staining, MT staining, RT-qPCR, IHC, WB, and IF analysis.

#### Statistical analysis

Statistical analysis was performed using SPSS software (version 27.0) and GraphPad Prism (version 10.0). The normality of data distribution was assessed using the Shapiro–Wilk test. Normally distributed data were presented as mean ± standard deviation (SD), while non-normally distributed data were expressed as median (interquartile range, IQR). For comparisons between two groups, a two-tailed Student’s *t*-test (normal distribution) or Mann–Whitney U test (non-normal distribution) was used. For comparisons among multiple groups, one-way analysis of variance (ANOVA) followed by Tukey’s post hoc test or the Kruskal–Wallis test was applied as appropriate. For experiments involving two independent variables (e.g. treatment and time factors), two-way ANOVA was employed. Specifically, longitudinal wound healing data were analyzed using two-way repeated measures ANOVA followed by Bonferroni’s post hoc test. Differences were considered statistically significant at *P* < 0.05.

## Results

### Bioinformatics analysis of dysregulated RNA-binding proteins in fibroblasts from diabetic foot ulcer

**Figure 1:**
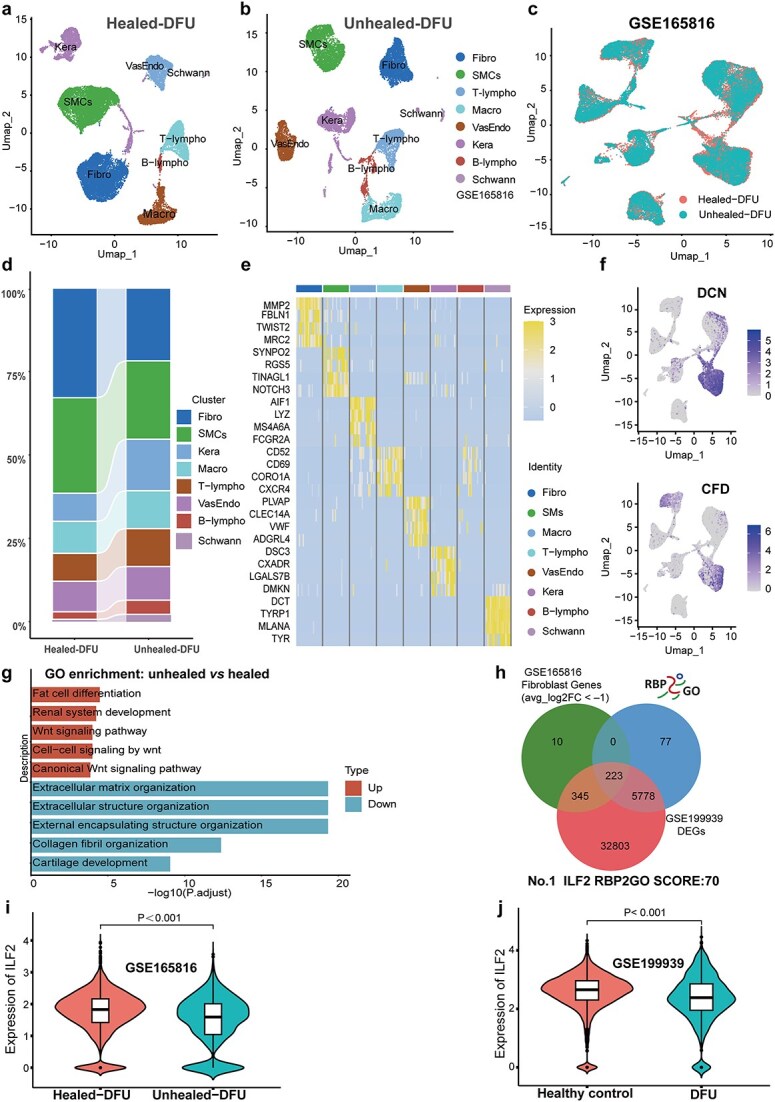
Bioinformatics analysis of dysregulated RBPs in fibroblasts from DFU. (**a**–**c**) tSNE plots showing cell clusters in healed DFU (a), unhealed DFU (b), and combined samples (c). Cells from different patients are grouped together. Distinct cell types are indicated by different colors. (**d**) Sankey plot based on the GSE165816 scRNA-seq dataset. (**e**) Heatmap of representative marker gene expression across cell types, highlighting distinct transcriptional profiles of each cell population. (**f**) UMAP plots showing the expression of fibroblast-related marker genes DCN and CFD across all cell populations. (**g**) GO enrichment analysis of genes from fibroblast cells in healed and unhealed DFU samples. (**h**) Venn diagram showing the overlap between RBP2GO dataset and DEGs from GSE199939 and fibroblast clusters (avg_log2FC < −1) in GSE165816. (**i**) ILF2 expression in fibroblast cells from healed and unhealed DFU tissues within fibroblast clusters from GSE165816. (**j**) ILF2 expression in DFU and normal tissues based on transcriptome data from GSE199939. *DFU* Diabetic foot ulcer

To investigate cellular heterogeneity and transcriptional alterations associated with DFU healing status, we analyzed single-cell RNA-seq (scRNA-seq) data from healed and non-healed DFU tissues (GSE165816) [27]. UMAP projections (Figure 1a and b) revealed distinct clusters of fibroblasts, immune cells, and other cell types, clearly delineated by canonical marker genes. Notably, cells derived from different patients exhibited comparable clustering patterns. A joint UMAP analysis of both conditions (Figure 1c) confirmed transcriptional divergence among fibroblasts, demonstrating clear segregation between healed and non-healed states. A Sankey diagram (Figure 1d) further illustrated cell type distribution across healing states, emphasizing shifts in fibroblast populations and their relative abundance. Meanwhile, fibroblasts play a crucial role in wound healing, and maintaining their integrity is essential for successful tissue repair. Therefore, we focus on the functions and behaviors of fibroblasts in the context of tissue regeneration.

Marker gene expression heatmaps (Figure 1e) highlighted pronounced differences in transcriptional profiles of cell subtypes between healed and non-healed DFUs. Furthermore, we visualized the expression patterns of fibroblast markers DCN and CFD across all cell populations (Figure 1f) [27], corroborating the accuracy of cell-type classification in the UMAP projection. GO enrichment analysis (Figure 1g) revealed that fibroblasts from non-healed DFU were enriched in pathways related to extracellular matrix organization and immune regulation, whereas fibroblasts in healed DFUs exhibited enrichment in pathways linked to cell signaling and tissue development. Research indicates [12–14] that senescent fibroblasts significantly impair skin wound healing by secreting the SASP and disrupting tissue homeostasis, this results in impaired cell proliferation, reduced collagen production, and an imbalance between extracellular matrix synthesis and degradation, further hindering wound repair, consistent with the results of GO analyses. Given the central role of fibroblasts in wound repair, these findings suggest that impaired fibroblast function may critically contribute to delayed wound healing in diabetic ulcers.

RBPs [16] are key regulators of RNA stability, splicing, transport, and translation, influencing critical biological processes like immune response, cellular stress, metabolism, and tumor igenesis. To identify dysregulated RBPs in DFU fibroblasts, we conducted an integrative analysis combining DEGs from the bulk transcriptomic dataset GSE199939 and fibroblast-specific DEGs from the GSE165816 single-cell dataset. These were intersected with the human RBP dataset from RBP2GO (https://rbp2go.dkfz.de/) [29]. Venn analysis (Figure 1h) revealed 223 downregulated candidate regulatory RBPs common to both datasets, with ILF2 (Score:70) being the highest-ranked in the RBP2GO prioritization. Single-cell expression analysis in GSE165816 showed significantly reduced ILF2 expression in fibroblasts from unhealed-DFU (Figure 1i). Consistently, bulk RNA-seq data from GSE199939 demonstrated lower ILF2 expression in DFU tissues compared to healthy control (Figure 1j). These results suggest that the downregulation of ILF2 in fibroblasts plays a critical role in the pathogenesis of delayed wound healing in DFU.

### Interleukin enhancer-binding factor 2 expression is downregulated in human diabetic foot ulcer tissues, diabetic mice, and high glucose–treated cells

**Figure 2:**
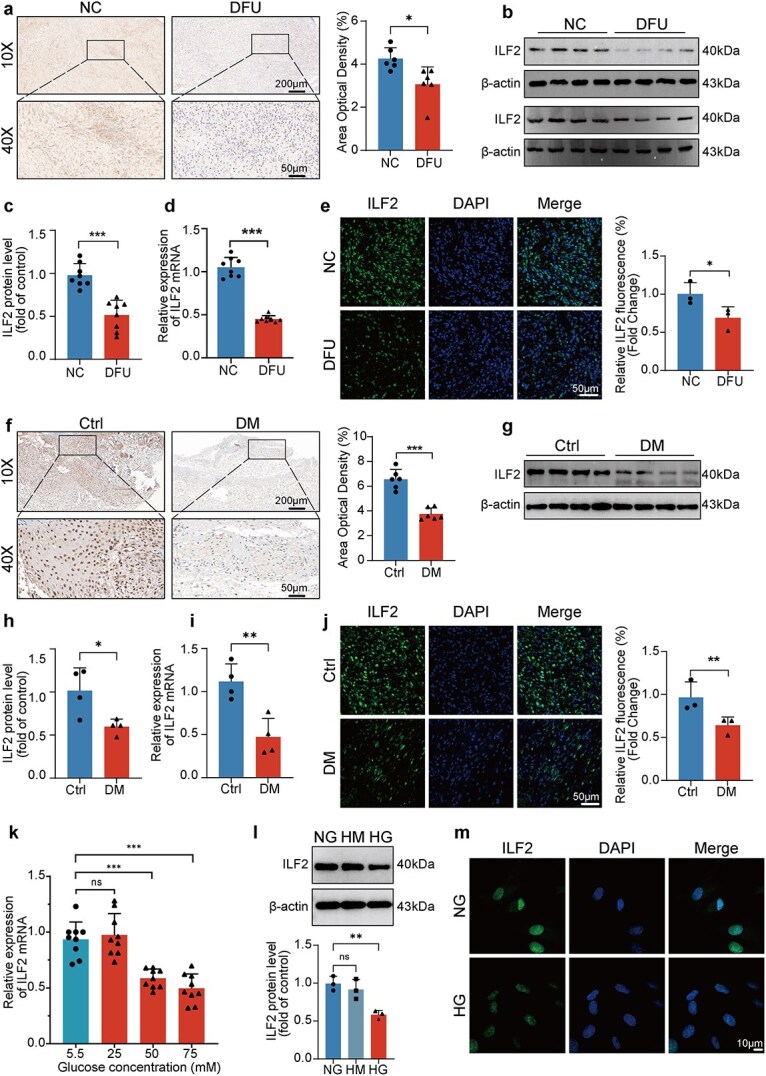
ILF2 expression is downregulated in human DFU tissues, diabetic mice, and HG-treated cells. (**a**) Representative immunohistochemical staining of ILF2 in wound tissues from non-diabetic trauma patients (NC) and diabetic foot ulcer (DFU) patients at low (10×) and high (40×) magnifications. Scale bars, 200 (upper) and 50 μm (lower). (**b**-**c**) Western blot analysis of ILF2 expression in wound tissues from NC and DFU patients.(**d**) ILF2 mRNA expression levels in wound tissues from NC and DFU patients.(**e**) Representative immunofluorescence staining of ILF2 (green) and nuclei (DAPI, blue) in wound tissues from NC and DFU patients (scale bar: 50 μm).(**f**-**j**) Similar analyses in skin wound tissues from control (Ctrl) and diabetic (DM) mice, including IHC (f), western blot (g-h), qPCR (i) and IF (j). (**k**) qPCR analysis of ILF2 expression in cells cultured under varying glucose concentrations (5.5, 25, 50, and 75 mM). (**l**) Western blot analysis of ILF2 expression in cells cultured under NG (5.5 mM), HM, and HG (50 mM) conditions. (**m**) IF staining of ILF2 (green) and nuclei (DAPI, blue) in cells cultured under NG and HG conditions (scale bar: 10 μm). Ns, not significant; *P* > 0.05; ^*^P < 0.05; ^**^P < 0.01; ^***^P < 0.001. *DM* diabetes mellitus, *IHC* immunohistochemistry, *qPCR* quantitative polymerase chain reaction; *DAPI* 4′,6-diamidino-2-phenylindole

To evaluate aberrant expression of the RNA-binding protein ILF2 in DFU tissues, we performed IHC staining on wound samples from DFU patients and non-diabetic chronic lower extremity ulcer (NC) patients with trauma. IHC staining and quantitative analysis revealed significantly lower ILF2 expression in DFU tissues compared to NC (Figure 2a). Quantitative PCR confirmed reduced ILF2 mRNA levels in DFU samples (Figure 2b, Figure S2b). Consistently, immunofluorescence (IF) staining revealed weaker ILF2 signals in DFU tissues, accompanied by reduced fluorescence intensity (Figure 2c). Western blot analysis further validated ILF2 downregulation in DFU skin (Figure 2d, Figure S2a). Similar results were obtained in diabetic (DM) mice, where ILF2 expression was consistently decreased in wound tissues as demonstrated by IHC, qPCR, immunofluorescence, and Western blot assays (Figure 2e–h).

To investigate the expression of ILF2 in an *in vitro* diabetic model, fibroblasts were treated with different glucose concentrations (5.5, 25, 50, and 75 mM). qPCR analysis showed a dose-dependent decrease in ILF2 expression, with a significant reduction at 50 mM (Figure 2i). Therefore, we selected 50 mM glucose as the intervention concentration. Western blot analysis further confirmed the downregulation of ILF2 under HG (50 mM) conditions (Figure 2j). Compared to normal glucose (NG) and HM conditions, immunofluorescence staining revealed reduced ILF2 fluorescence intensity in cells treated with HG (Figure 2k). In summary, both *in vivo* and *in vitro* experiments confirmed that ILF2 expression is significantly downregulated in DFU tissues, highlighting its potential role in the disease’s pathogenesis.

### Diabetic wounds exhibit delayed healing and a senescence-associated secretory phenotype

**Figure 3:**
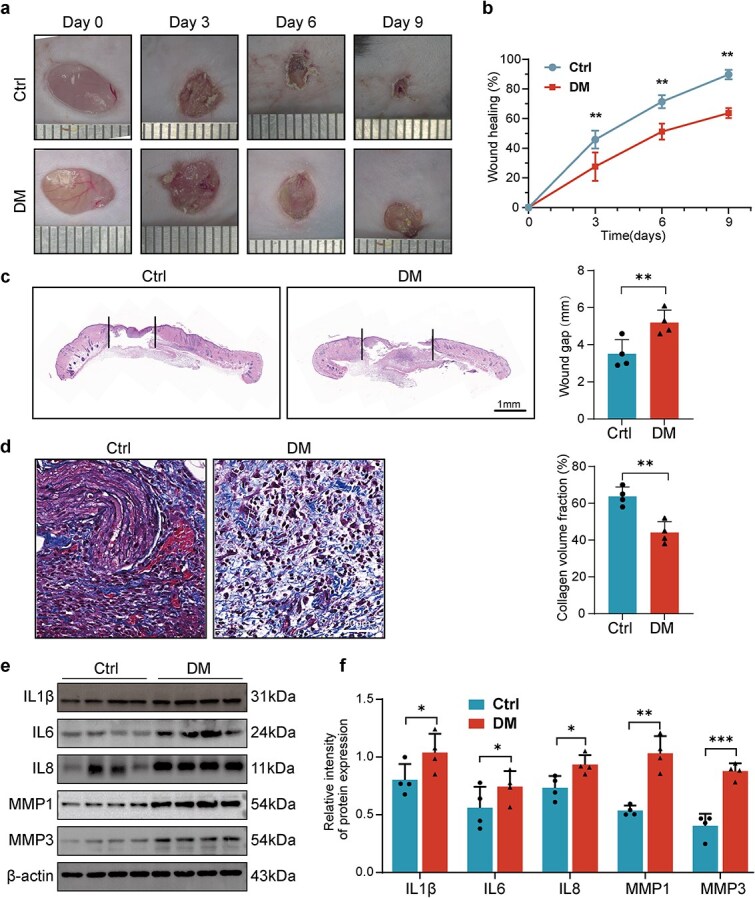
Diabetic wounds exhibit delayed healing and a SASP. (**a**) Representative images of wound areas in diabetic (DM) and control (Ctrl) mice at the indicated time points (Day 0, Day 3, Day 6, and Day 9). (**b**) Quantification of wound healing in DM and Ctrl mice, expressed as the percentage of the initial wound area (*n* = 4). (**c**) H&E staining of Day-6 skin wound tissues (scale bar: 1 mm). (**d**) Masson’s trichrome staining of Day-6 skin wound tissues (scale bar:100 μm). (**e** and **f**) representative western blot analysis of SASP factors (IL1β, IL6, IL8, MMP1, and MMP3) in skin wound tissues from control and DM mice, with β-actin as the loading control. ^*^P < 0.05; ^**^P < 0.01; ^***^P < 0.001. *SASP* senescence-associated secretory phenotype, *H&E* hematoxylin and eosin, *IL1β* interleukin-1β, *IL6* interleukin-6, *IL8* interleukin-8, *MMP1* matrix metalloproteinase-1, *MMP3* matrix metalloproteinase-3

To compare the differences in wound healing between diabetic and normal wounds, we created 6-mm full-thickness wounds on the dorsal skin of diabetic/non-diabetic mice. As shown in Figure 3a and b, the wound healing process was slower in diabetic mice compared to the control group. Notably, the wound area of diabetic mice was significantly larger than that of the control group on Day 3 (*P* < 0.05), Day 6 (*P* < 0.05), and Day 9 (*P* < 0.001). Even after 9 days of healing, diabetic mice still exhibited noticeably larger wounds, whereas the wounds in the control group had completely healed. Therefore, diabetic mice demonstrated prolonged wound closure time. Additionally, H&E staining revealed wider gaps in the skin of diabetic mice (Figure 3c). Masson’s trichrome staining (Figure 3d) showed a significant reduction in collagen deposition and disorganized collagen fiber alignment in the diabetic mouse skin wound tissue. One of the well-known markers of fibroblast senescence [30] the SASP factors the list of SASP factors is summarized in Table S6 [8], typical SASP factors include IL-1β, IL-6, IL-8, MMP1, and MMP-3. Western blot analysis confirmed the sustained high-level expression of SASP in diabetic wounds (Figure 3e and f). In summary, these findings suggest that senescent cells persistently accumulate in diabetic wounds, contributing to impaired wound healing.

### Interleukin enhancer-binding factor 2 suppresses senescence-associated secretory phenotype factor expression while promoting fibroblast proliferation and migration

**Figure 4:**
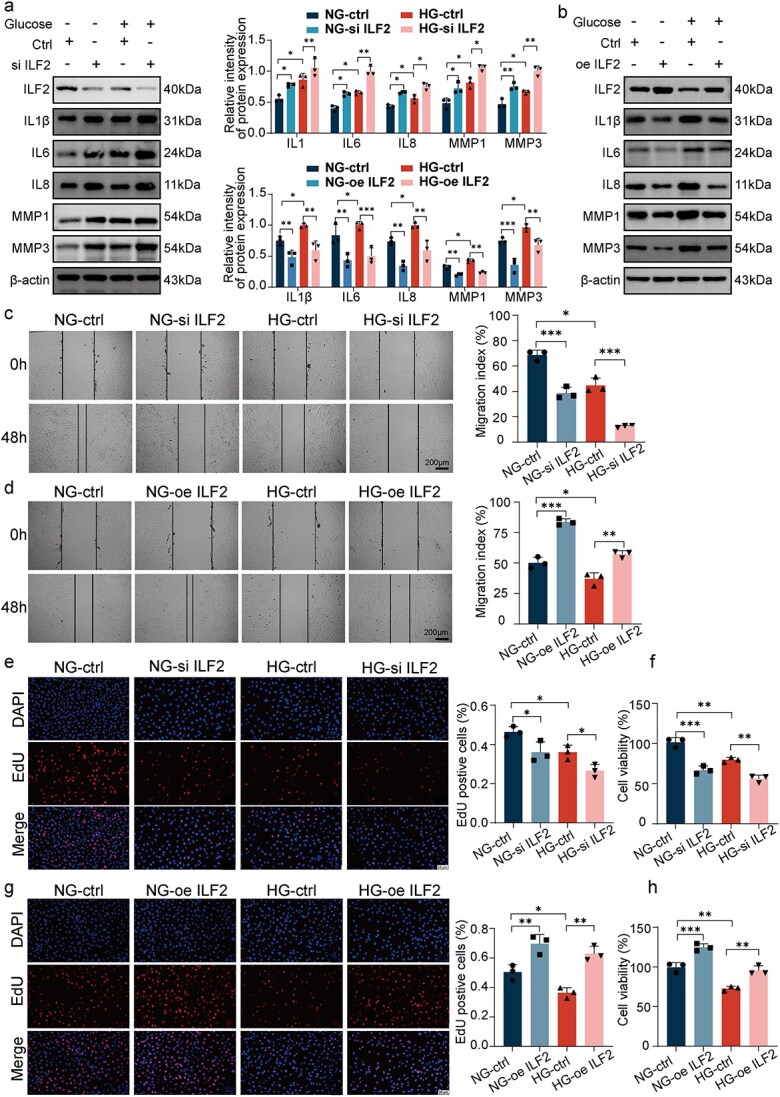
ILF2 suppresses SASP factor expression while promoting fibroblast proliferation and migration under NG and HG conditions. (**a** and **b**) western blot analysis of ILF2 knockdown (siILF2) or overexpression (oeILF2) under NG and HG conditions, showing the expression levels of SASP factors (IL-1β, IL-6, IL-8, MMP1, and MMP3). (**c** and **d**) Wound healing assays of fibroblasts under NG and HG conditions following ILF2 knockdown or overexpression (scale bar: 200 μm). (**e** and **g**) EdU incorporation assays were performed to evaluate cell proliferation in fibroblasts under NG and HG conditions following ILF2 knockdown (e) or overexpression (g). EdU-positive proliferating cells are shown in red, with nuclei counterstained by DAPI (scale bar: 50 μm). (**f** and **h**) CCK-8 assays were conducted to assess proliferative activity of fibroblasts under NG and HG conditions after ILF2 knockdown (f) or overexpression (h). ^*^P < 0.05; ^**^P < 0.01; ^***^P < 0.001. *SASP* senescence-associated secretory phenotype, *CCK-8* cell counting Kit-8, *IL* interleukin, *MMP1* matrix metalloproteinase-1

Given the downregulation of ILF2 in DFU, we investigated its functional role in fibroblasts under diabetic-like conditions. To this end, ILF2 expression was modulated via siRNA-mediated knockdown and plasmid-mediated overexpression in fibroblasts cultured under both NG (5.5 mM) and HG (50 mM) conditions. Western blot analysis revealed that ILF2 knockdown significantly upregulated the expression of SASP factors, including IL1β, IL6, IL8, MMP1, and MMP3, particularly under HG conditions. Conversely, ILF2 overexpression suppressed the expression of these SASP factors (Figure 4a and b). Additionally, wound healing assays demonstrated that ILF2 overexpression enhanced fibroblast migration, as shown by faster wound closure, while ILF2 knockdown impaired wound healing under both NG and HG conditions (Figure 4c and d). Cell proliferation assays further validated these findings. EdU-positive cells revealed that ILF2 overexpression enhanced fibroblast proliferation, while ILF2 knockdown inhibited proliferation under both NG and HG conditions (Figure 4e and g). These results were further corroborated by CCK-8 assays (Figure 4f and h). Collectively, these results suggest that ILF2 inhibits HG-induced SASP in fibroblasts and promotes their proliferation and migration.

### RNA sequencing and bioinformatics analysis identify nucleophosmin as a direct target of interleukin enhancer-binding factor 2

**Figure 5:**
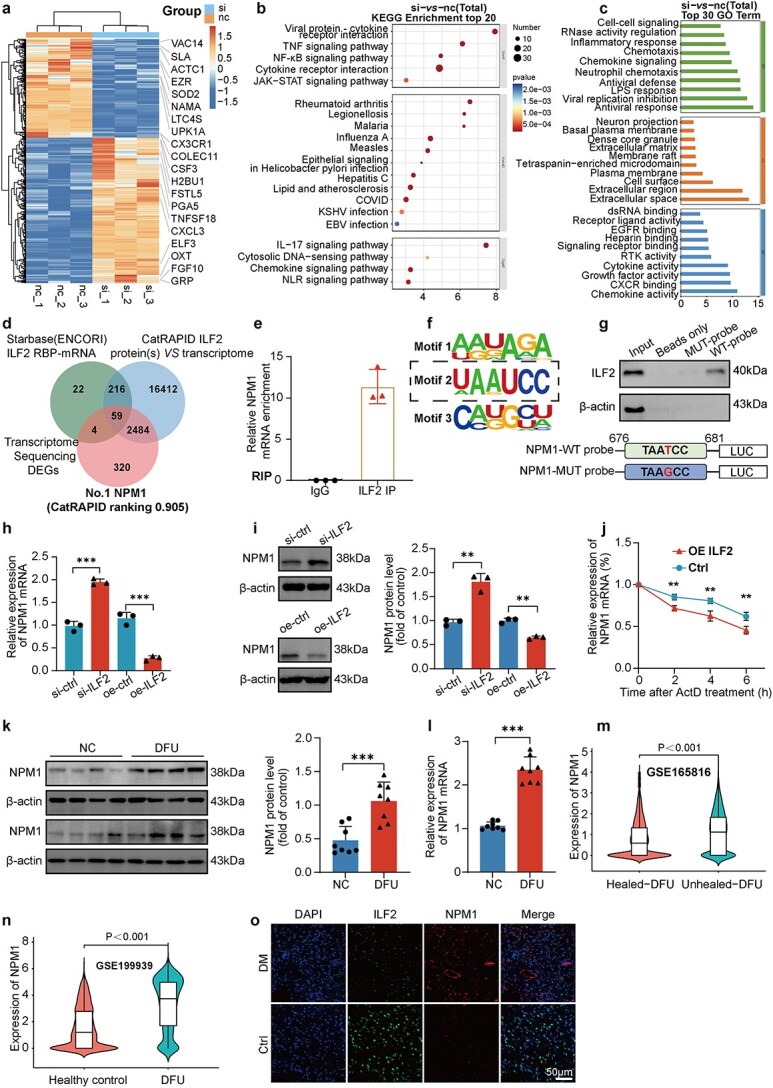
RNA-seq and bioinformatics analysis identify NPM1 as a direct target of ILF2. (**a**) Heatmap showing DEGs following ILF2 knockdown in fibroblasts. (**b** and **c**) KEGG pathway enrichment analyses of ILF2-regulated transcriptomic changes. (**d**) Venn diagram showing the overlap among predicted ILF2-binding mRNAs from ENCORI and catRAPID databases and DEGs from transcriptome sequencing. The top candidate identified within the overlap by catRAPID ranking was NPM1. (**e**) RIP assay of ILF2 protein with NPM1 mRNA. (**f**) Identification of the top three RNA motif sequences associated with ILF2 binding using the RBP-MotifBrowse function in starBase (ENCORI). (**g**) Western blot analysis revealed that only the Motif2 NPM1 mRNA probe effectively pulled down ILF2 protein, validating their direct interaction. (**h**) qRT-PCR analysis of NPM1 mRNA expression following ILF2 knockdown or overexpression in fibroblasts. (**i**) Western blot analysis of NPM1 protein levels after ILF2 knockdown or overexpression in fibroblasts. (**j**) mRNA stability assay of NPM1 following ILF2 overexpression in fibroblasts, assessed after actinomycin D treatment. (**k**) Western blot analysis of NPM1 expression in wound tissues from DFU patients and non-diabetic trauma patients (NC). (**l**) RT-PCR analysis of NPM1 expression in wound tissues from DFU patients and NC. (**m** and **n**) NPM1 expression in fibroblast clusters from unhealed and healed DFU tissues based on scRNA-seq data (GSE165816), and in DFU versus healthy skin samples based on bulk transcriptomic data (GSE199939). (**o**) Immunofluorescence staining of wound tissues from diabetic (DM) and control (Ctrl) mice showing ILF2 and NPM1 expression (scale bar: 50 μm). ^*^P < 0.05; ^**^P < 0.01; ^***^P < 0.001. qRT-PCR, quantitative real-time polymerase chain reaction, *DFU* diabetic foot ulcer, *DM* diabetes mellitu, *ILF2* interleukin enhancer-binding factor 2, *NPM1* nucleophosmin 1

Considering the role of ILF2 in improving inflammatory senescence in fibroblasts, we further explored its potential mechanisms. To simulate a diabetic environment, we performed RNA-seq (si ctrl vs si ILF2) on fibroblasts cultured under HG conditions to identify potential downstream targets of ILF2 at the transcriptional level. RNA-seq analysis revealed 1450 upregulated and 1417 downregulated genes. The heatmap of DEGs showed that ILF2 knockdown under HG conditions induced substantial transcriptional changes (Figure 5a). KEGG pathway analysis indicated that the DEGs were predominantly enriched in pathways related to inflammation and cellular stress (Figure 5b), whereas GO analysis highlighted biological processes associated with cellular function and immune responses (Figure 5c).

RBPs exert their functions through various molecular mechanisms, with the regulation of mRNA stability being one of their core roles [31]. Meanwhile, the CatRAPID [32] database shows that ILF2 contains a single RNA-binding domain, enabling it to interact with mRNA, as evidenced by its detection in RNA-binding motifs (Figure S1a–d). To explore potential mRNAs interacting with ILF2, we intersected the RBP–mRNA dataset from starBase (ENCORI) [33], the catRAPID [protein(s) vs transcriptome] dataset [32], and the DEGs from ILF2 knockdown fibroblast RNA-seq dataset. This three-way integration identified 59 potential mRNA targets of ILF2 (Figure 5d). Subsequently, we ranked these targets based on their interaction scores from the catRAPID database, where NPM1 showed the highest score (0.905), suggesting it may be a key target of ILF2.Subsequently, we preliminarily verified the interaction between ILF2 and NPM1 mRNA through RNA immunoprecipitation (RIP) (Figure 5e). To further identify the specific binding sites of ILF2 on NPM1 mRNA, we utilized the RBP-MotifBrowse function in the starBase (ENCORI) database and screened out the top three (rank 1–3) RNA motif sequences (Motif1, Motif2, and Motif3) associated with ILF2 binding, based on the statistical significance of log₁₀ (pval) and the target enrichment of Target (%) (Figure 5f). Based on these motifs, we designed RNA probes targeting the Motif1, Motif2, and Motif3 regions of NPM1 mRNA, respectively, and performed RNA Pull-down assays. Western blot analysis showed that only the RNA probe targeting the Motif2 region effectively pulled down ILF2 protein (Figure 5g), whereas the probes targeting the Motif1 and Motif3 regions did not detect ILF2 binding (Figure S1e). This result further confirmed the direct binding between ILF2 and NPM1 mRNA.

Next, we investigated the relationship between ILF2 and NPM1 expression levels. Consistent with the RNA-seq results, RT-qPCR, Western blot, and immunofluorescence experiments validated that ILF2 negatively regulates NPM1 expression (Figure 5h and i, Figure S1f). Specifically, ILF2 knockdown increased NPM1 protein expression, whereas ILF2 overexpression reduced its abundance. To further elucidate the mechanism by which ILF2 regulates NPM1 expression, we used the transcription inhibitor actinomycin D to assess NPM1 mRNA stability. RT-qPCR results showed that the half-life of NPM1 mRNA was approximately 6 h in control (ctrl) fibroblasts, but was significantly shortened to about 4 h in ILF2-overexpressing (oeILF2) cells (*P* < 0.05) (Figure 5j). Crucially, to validate the clinical relevance of these findings, we performed RIP, RNA pull-down (using the Motif2 probe), and mRNA stability assays using primary fibroblasts isolated from three DFU patients (Figure S3c–f). Consistent with the results in cell lines, these experiments confirmed the direct interaction between ILF2 and NPM1 mRNA and demonstrated that ILF2 negatively regulated NPM1 mRNA stability in DFU primary fibroblasts. Taken together, these findings indicate that ILF2 reduces the stability of NPM1 mRNA by directly binding to it, ultimately leading to the downregulation of NPM1 protein expression.

Previous studies have demonstrated that NPM1 is highly expressed in various pathological conditions, where it drives inflammation [34, 35], impairs tissue repair [36], and promotes cellular senescence [37]. To assess its relevance in DFUs, we analyzed public datasets (GSE165816 and GSE199939, Figure 5m and n) and found that NPM1 was consistently upregulated in DFU. This upregulation was further validated by qPCR, WB (Figure 5k and l), and IF (Figure S1g). Consistent results were also obtained in diabetic (DM) mouse wound tissues, where dual immunofluorescence analysis demonstrated downregulation of ILF2 accompanied by upregulation of NPM1 (Figure 5o). Collectively, These findings suggest that ILF2 binds to NPM1 mRNA and promotes its degradation, thereby regulating NPM1 expression, which may play a crucial role in the pathogenesis of DFU.

ILF2 inhibits the expression of SASP and promotes fibroblast proliferation and migration by regulating NPM1 expression.

Given that ILF2 regulates mRNA stability and subsequently affects NPM1 abundance, we aimed to validate NPM1 as a potential regulatory factor in the downstream progression of ILF2-mediated inflammatory senescence.

**Figure 6:**
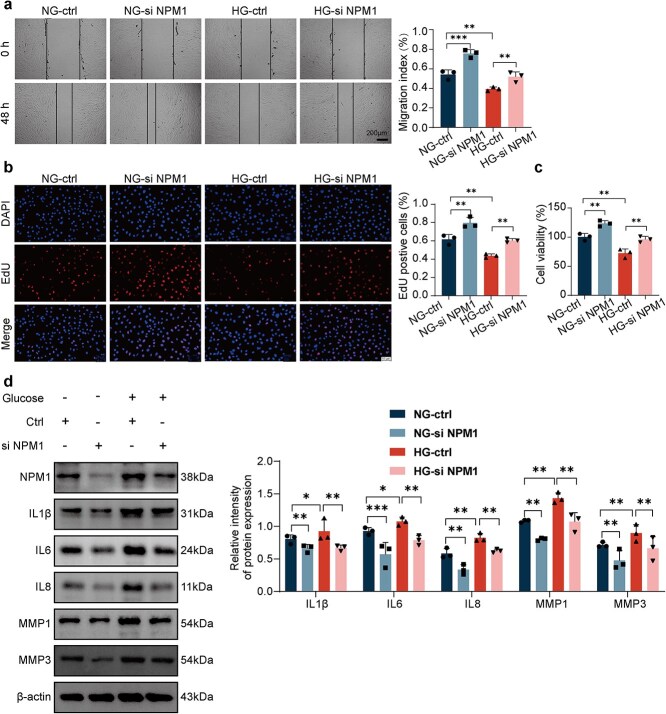
NPM1 promotes SASP factor expression while suppressing fibroblast proliferation and migration under NG and HG conditions. (**a**) Wound healing assays of fibroblasts cultured under NG and HG conditions following NPM1 knockdown (scale bar: 200 μm). (**b**) EdU assays to assess cell proliferation under NG and HG conditions following NPM1 knockdown. EdU-positive cells are shown in red, and nuclei were counterstained with DAPI (scale bar: 50 μm). (**c**) CCK-8 assays evaluating proliferative activity under NG and HG conditions following NPM1 knockdown. (**d**) Western blot analysis of SASP factors (IL-1β, IL-6, IL-8, MMP1, and MMP3) in fibroblasts under NG and HG (50 mM) conditions after NPM1 knockdown. ^*^P < 0.05; ^**^P < 0.01; ^***^P < 0.001. *SASP* senescence-associated secretory phenotype, *NG* normal glucose, *HG* high glucose, *DAPI* 4’,6-diamidino-2-phenylindole, *IL* interleukin, *MMP1* matrix metalloproteinase-1*CCK-8* cell counting Kit-8

To independently assess the function of NPM1, we performed siRNA-mediated knockdown of NPM1 in fibroblasts cultured under NG, 5.5 mM or HG conditions. Consistent with previous observations, HG treatment increased NPM1 expression. Functional assays revealed that NPM1 knockdown significantly enhanced fibroblast proliferation and migration under both NG and HG conditions, as demonstrated by CCK-8, EdU, and wound healing assays (Figure 6a–c). Furthermore, Western blot analysis showed that NPM1 knockdown reduced the expression of SASP factors, including IL1β, IL6, IL8, MMP1, and MMP3 (Figure 6d). Overall, NPM1 promotes fibroblast senescence while suppressing their proliferation and migration.

**Figure 7:**
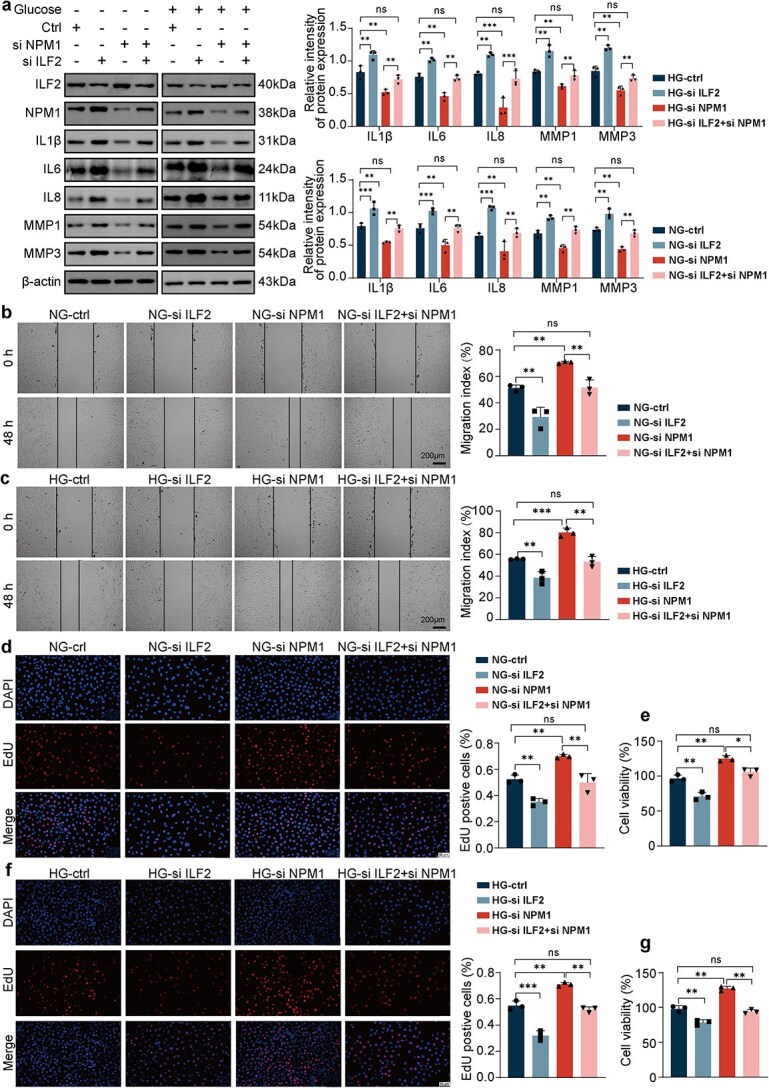
ILF2 inhibits the expression of SASP and promotes fibroblast proliferation and migration by regulating NPM1 expression. (**a**) Western blot analysis of ILF2, NPM1, and SASP factors (IL-1β, IL-6, IL-8, MMP1, and MMP3) in fibroblasts co-transfected with siILF2 and siNPM1 under NG and HG conditions. (**b**, **c**) wound healing assays showing cell migration under NG and HG conditions with siILF2, siNPM1, or co-transfection treatments(scale bar: 200 μm). (**d** and **f**) EdU assays to assess cell proliferation under NG and HG conditions following siRNA treatments. EdU-positive cells are shown in red, and nuclei are counterstained with DAPI (scale bar: 50 μm). (**e** and **g**) CCK-8 assays to evaluate proliferative activity under NG and HG conditions following siRNA treatments. Ns, not significant; *P* > 0.05; ^*^P < 0.05; ^**^P < 0.01; ^***^P < 0.001. *SASP* senescence-associated secretory phenotype, *NG* normal glucose, *HG* high glucose, EdU 5-ethynyl-2’-deoxyuridine, *DAPI* 4’,6-diamidino-2-phenylindole, *CCK-8* Cell Counting Kit-8, *IL* interleukin, *MMP1* matrix metalloproteinase-1

To further elucidate the functional relationship between ILF2 and NPM1, we performed rescue experiments in fibroblasts by simultaneously applying siRNA-mediated knockdown of both genes under NG and HG conditions. Western blot analysis showed that NPM1 knockdown reduced the elevated expression of SASP factors (IL1β, IL6, IL8, MMP1, and MMP3) caused by ILF2 knockdown (Figure 7a). Consistently, NPM1 knockdown restored the impaired migratory capacity induced by ILF2 knockdown, as demonstrated by wound healing assays (Figure 7b and c). Furthermore, CCK-8 and EdU assays confirmed that NPM1 knockdown significantly rescued the proliferation defect of ILF2-deficient fibroblasts (Figure 7d–g). Notably, these rescue effects were observed under both NG and HG conditions. Taken together, these results indicate that ILF2 regulates fibroblast SASP expression, proliferation, and migration by modulating NPM1 expression.

### Interleukin enhancer-binding factor 2 and nucleophosmin regulate nuclear factor-kappa B signaling pathway and p65 activation in fibroblasts

**Figure 8:**
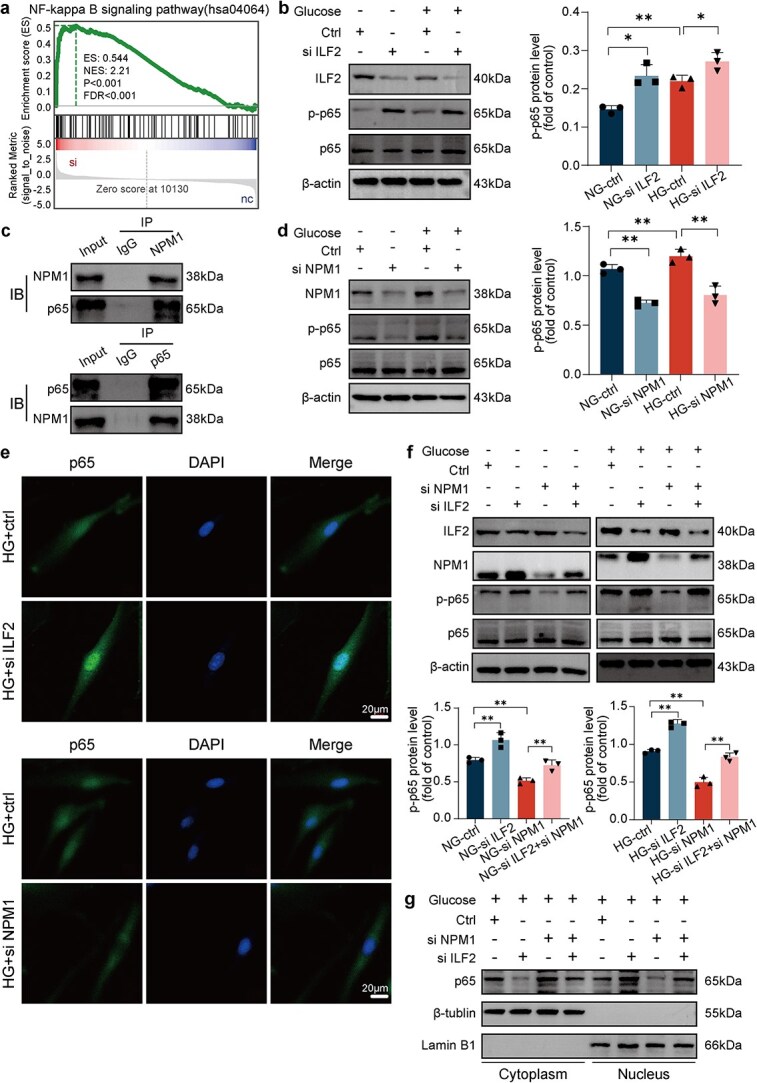
ILF2 and NPM1 regulate NF-κB signaling and p65 activation in fibroblasts. (**a**) GSEA of NF-κB signaling in the indicated dataset. (**b**) Western blot analysis of p-p65 in fibroblasts under NG and HG conditions after ILF2 knockdown. (**c**) Coimmunoprecipitation showing interaction between NPM1 and p65 in the indicated cells. (**d**) Western blot analysis of p-p65 in fibroblasts under NG and HG conditions after NPM1 knockdown. (**e**) Upper panel: p65 (green) and nuclei (DAPI, blue) immunofluorescence in HG + ctrl and HG + siILF2 fibroblasts (scale bar: 20 μm); lower panel: p65 (green) and nuclei (DAPI, blue) immunofluorescence in HG + ctrl and HG + siNPM1 fibroblasts (scale bar: 20 μm). (**f**) Western blot analysis of p-p65 in fibroblasts co-transfected with siILF2 and siNPM1 under NG and HG conditions. (**g**) Level of p65 detected by western blot in cytoplasmic and nuclear extraction. Β-tubulin was used as a cytoplasmic internal reference, and Lamin B1 was used as a nuclear internal reference. ^*^P < 0.05; ^**^P < 0.01; ^***^P < 0.001. NF-κB, nuclear factor kappa B, *GSEA* Gene Set Enrichment Analysis, *NG* normal glucose, *HG* high glucose, *DAPI* 4’,6-diamidino-2-phenylindole, *NPM1* nucleophosmin 1

Gene set enrichment analysis (GSEA) analysis revealed a significant enrichment of the NF-κB signaling pathway in ILF2-deficient fibroblasts. The enrichment plots showed that pathway-associated genes were predominantly concentrated in the upregulated region, indicating that the loss of ILF2 activates NF-κB signaling (Figure 8a). This finding is particularly significant given that NF-κB acts as the master regulator of the SASP, orchestrating the expression of a broad spectrum of pro-inflammatory factors, including IL-1α, IL-6, IL-8, and various chemokines (Wang *et al*., 2024) [8]. Consistently, Western blotting confirmed that ILF2 knockdown increased the expression of phospho-p65 (p-p65) (Figure 8b). Conversely, ILF2 overexpression notably decreased phospho-p65 levels, indicating inhibition of NF-κB pathway activation (Figure S1h). Notably, we validated these findings in primary fibroblasts, observing a consistent pattern of NF-κB regulation by ILF2 (Figure S3a and b).

Mechanistically, NPM1 was identified as a leading-edge gene in the NF-κB pathway, implicating it as a potential downstream mediator of ILF2. Previous studies [34, 35] have reported that NPM protein interacts with the NF-κB subunit p65. Supporting this link, Co-IP assays confirmed a direct interaction between NPM1 and p65 in fibroblasts (Figure 8c). Furthermore, NPM1 knockdown significantly reduced p65 phosphorylation (Figure 8d). In parallel, immunofluorescence analysis revealed that ILF2 knockdown enhanced p65 nuclear translocation in HG-treated fibroblasts, consistent with the observations of Rao *et al.* [34, 35]. In contrast, NPM1 knockdown attenuated this nuclear translocation (Figure 8e). Crucially, rescue experiments showed that NPM1 depletion abrogated the increase in p-p65 levels induced by ILF2 knockdown under both NG and HG conditions (Figure 8f). Subcellular fractionation assays further validated that NPM1 depletion prevented the ILF2 knockdown-induced nuclear accumulation of p65 (Figure 8g). Collectively, these findings suggest that ILF2 functions as a negative regulator of NF-κB signaling by suppressing NPM1, thereby inhibiting p65 phosphorylation and nuclear translocation to reduce HG-induced SASP expression in fibroblasts.

### Interleukin enhancer-binding factor 2 promotes wound healing in diabetic mice by targeting nucleophosmin

To investigate the role of ILF2 in wound healing in diabetic mice, we established a STZ-induced diabetic (DM) mouse model. Mice with fasting blood glucose levels ≥250 mg/dL (16.7 mmol/L) in two consecutive measurements were considered diabetic. AAV-shILF2 or AAV-oeILF2 vectors were subcutaneously injected into the dorsal region of diabetic mice for 4 weeks. The experimental groups included: normal control (non-sh ILF2-ctrl), normal knockdown (non-sh ILF2), diabetic control (DM-sh ILF2-ctrl), diabetic knockdown (DM-sh ILF2), normal overexpression (Non-oe ILF2-vector), normal overexpression (non-oe ILF2), diabetic control overexpression (DM-oe ILF2-vector), and diabetic overexpression (DM-oe ILF2).

**Figure 9:**
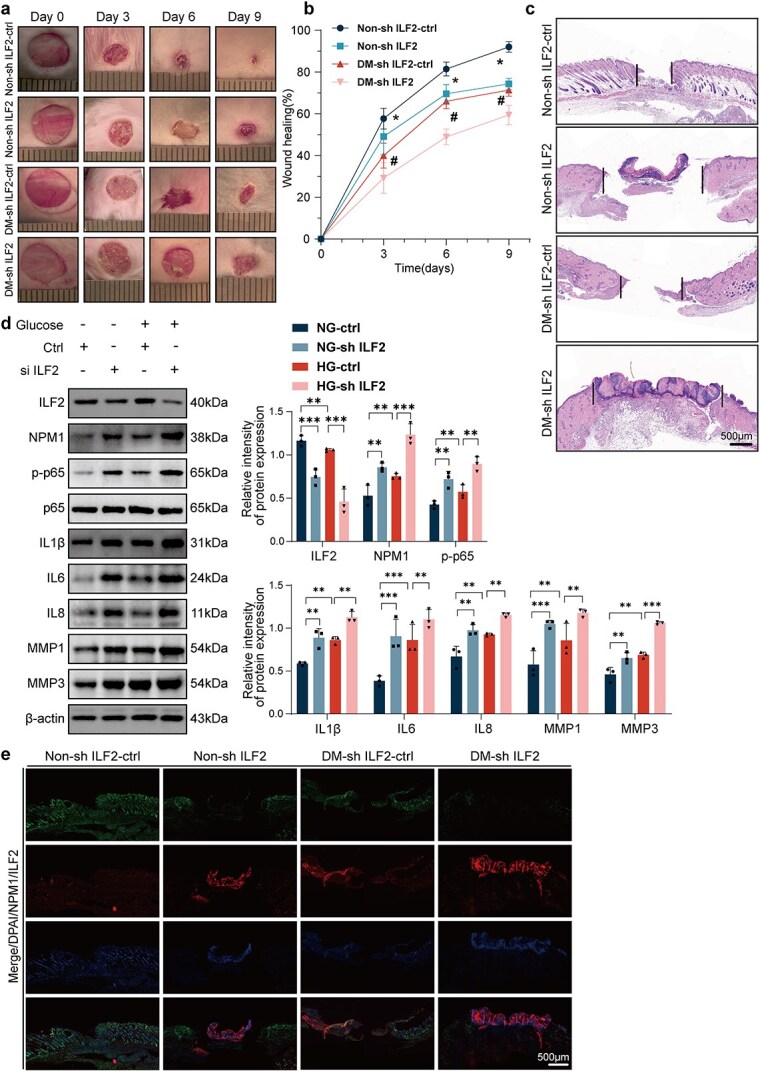
Knockdown of ILF2 delays wound healing and enhances inflammatory senescence in diabetic mice. (**a**) Representative images of wound closure at Days 0, 3, 6, and 9 in four groups: Non-sh ILF2-ctrl, non-sh ILF2, DM-sh ILF2-ctrl, and DM-sh ILF2. (**b**) Quantitative assessment of wound closure rate over time (*n* = 5 per group). (**c**) H&E-stained histological sections of skin wounds collected at Day 6. Scale bars: 500 μm. (**d**) Western blot analysis of ILF2, NPM1, total and phosphorylated p65 and SASP factors (IL-1β, IL-6, IL-8, MMP1, and MMP3) in wound tissues, with β-actin as a loading control. (**e**) Immunofluorescence staining of ILF2 (green), NPM1 (red), and nuclei (DAPI, blue) in wound tissues. Scale bars: 500 μm. ^*^P <0.05; ^**^P < 0.01; ^***^P < 0.001. *DM* diabetes mellitus, *H&E* hematoxylin and eosin, *SASP* senescence-associated secretory phenotype, *ILF2* interleukin enhancer-binding factor 2, *NPM1* nucleophosmin 1, *DAPI* 4’,6-diamidino-2-phenylindole, *IL* interleukin, *MMP1* matrix metalloproteinase-1

**Figure 10:**
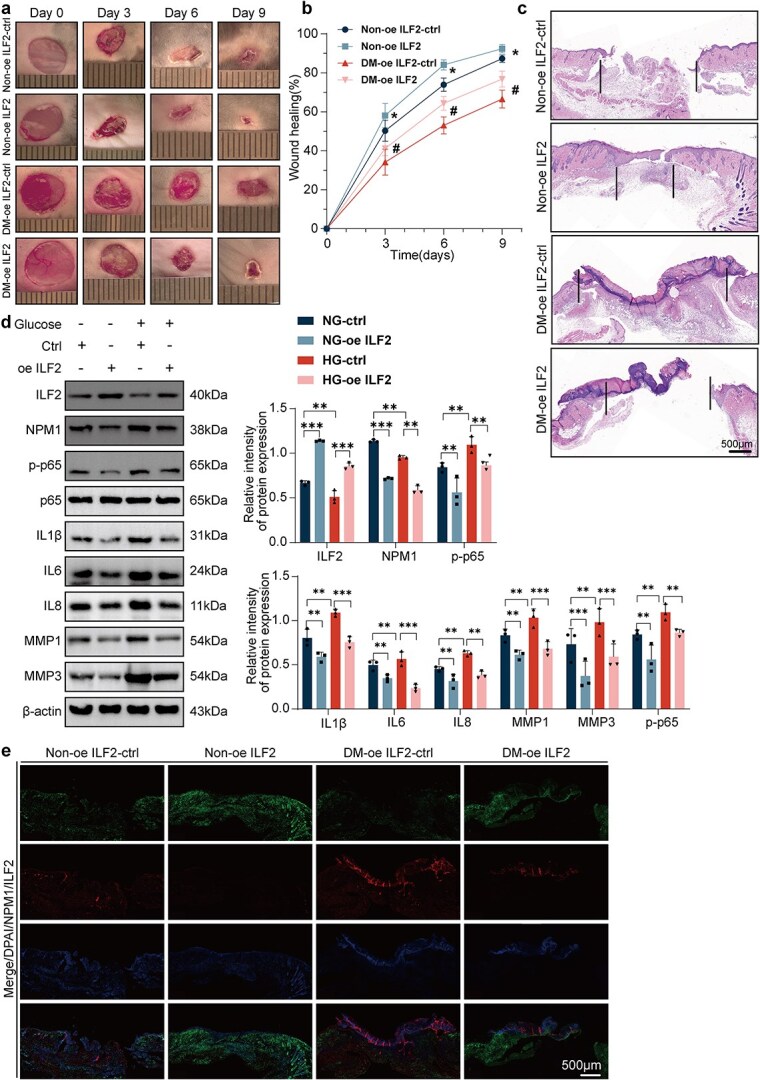
Overexpression of ILF2 accelerates wound healing and reduces inflammatory senescence in diabetic mice. (**a**) Representative images of wound closure at Days 0, 3, 6, and 9 in four groups: Non-oe ILF2-ctrl, non-oe ILF2, DM-oe ILF2-ctrl, and DM-oe ILF2. (**b**) Quantitative assessment of wound closure rate over time (*n* = 5 per group). (**c**) H&E-stained histological sections of skin wounds collected at Day 6. Scale bars: 500 μm. (**d**) Western blot analysis of ILF2, NPM1, total and phosphorylated p65 and SASP factors (IL-1β, IL-6, IL-8, MMP1, and MMP3) in wound tissues, with β-actin as a loading control. (**e**) Immunofluorescence staining of ILF2 (green), NPM1 (red), and nuclei (DAPI, blue) in wound tissues. Scale bars: 500 μm. ^*^P < 0.05; ^**^P < 0.01; ^***^P < 0.001. *DM* diabetes mellitus, *H&E* hematoxylin and eosin, *ILF2* interleukin enhancer-binding factor 2, *NPM1* nucleophosmin 1, *SASP* senescence-associated secretory phenotype, *DAPI* 4’,6-diamidino-2-phenylindole, *IL* interleukin, *MMP1* matrix metalloproteinase-1

ILF2 knockdown significantly delayed wound healing. The knockdown groups (non-sh ILF2, DM-sh ILF2) showed a significantly slower wound closure compared to the control groups (Figure 9a and b). In diabetic mice, the DM-sh ILF2 group exhibited the slowest healing, and histological analysis (Figure 9c) revealed larger skin fissures, indicating tissue damage and cell loss. Western blot analysis (Figure 9d) demonstrated that ILF2 knockdown increased NPM1 expression, elevated p65 phosphorylation levels, and upregulated the expression of SASP factors (IL-1β, IL-6, IL-8, MMP1, and MMP3), with more pronounced effects in diabetic mice. Immunofluorescence staining (Figure 9e) further supported that ILF2 negatively regulates NPM1.In contrast, ILF2 overexpression significantly accelerated wound healing, especially under HG conditions in diabetic mice. The DM-oe ILF2 group healed significantly faster than the DM-sh ILF2 group (Figure 10a and b). Histological analysis (Figure 10c) revealed that the ILF2 overexpression group exhibited a more intact wound tissue structure and smaller skin fissures, especially under HG conditions. These results further confirm ILF2’s role in promoting tissue repair in diabetic mice. Western blot analysis (Figure 10d) confirmed that ILF2 overexpression reduced NPM1 and p-p65 expression in diabetic wound tissue, and significantly decreased SASP factor expression (IL-1β, IL-6, IL-8, MMP1, and MMP3), especially under HG conditions. Immunofluorescence staining (Figure 10e) validated ILF2’s regulatory role in NPM1 expression.

**Figure 11:**
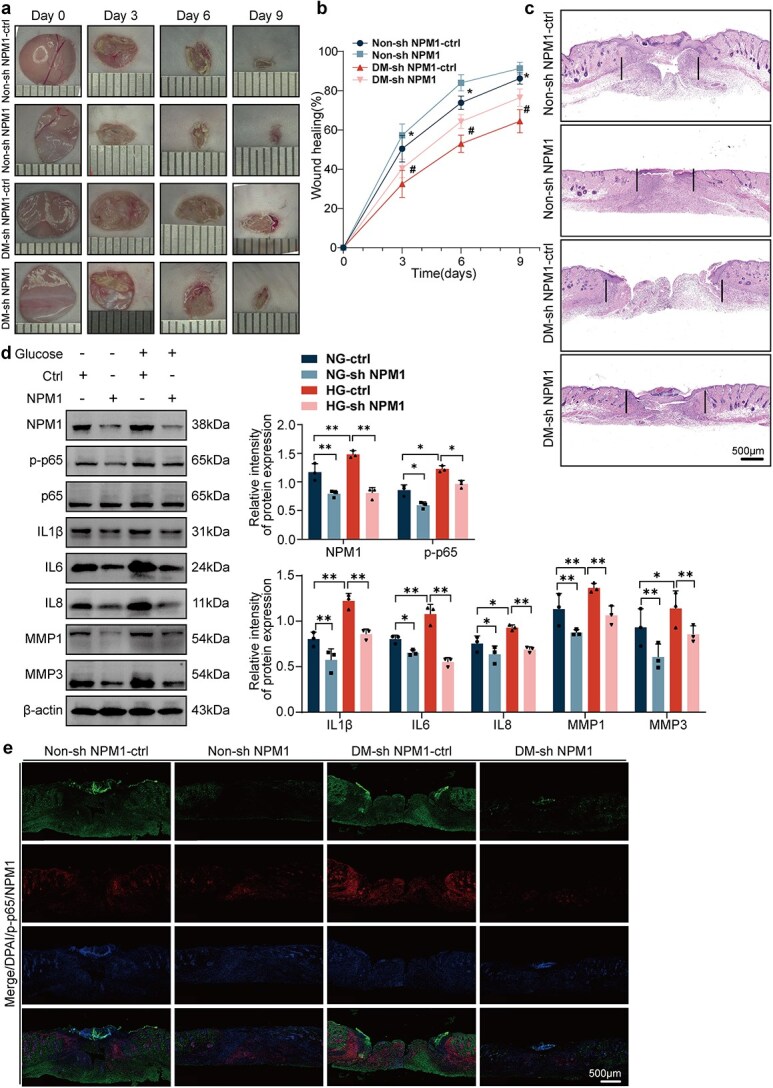
Knockdown of NPM1 accelerates wound healing and reduces inflammatory senescence in diabetic mice. (**a**) Representative images of wound closure at Days 0, 3, 6, and 9 in four groups: non-sh NPM1-ctrl, non-sh NPM1, DM-sh NPM1-ctrl, and DM-sh NPM1. (**b**) Quantitative assessment of wound closure rate over time (*n* = 5 per group). (**c**) H&E-stained histological sections of skin wounds collected at Day 6. Scale bars: 500 μm. (**d**) Western blot analysis of NPM1, total and phosphorylated p65 and SASP factors (IL-1β, IL-6, IL-8, MMP1, and MMP3) in wound tissues, with β-actin as a loading control. (**e**) Immunofluorescence staining of NPM1 (green), phosphorylated p65 (red), and nuclei (DAPI, blue) in wound tissues. Scale bars,: 500 μm. ^*^P < 0.05; ^**^P < 0.01; ^***^P < 0.001. *DM* diabetes mellitus, *H&E* hematoxylin and eosin, *ILF2* interleukin enhancer-binding factor 2, *NPM1* nucleophosmin 1, *SASP* senescence-associated secretory phenotype, *DAPI* 4’,6-diamidino-2-phenylindole, *IL* interleukin, *MMP1* matrix metalloproteinase-1

To further elucidate the downstream mechanism of ILF2, we investigated the functional role of NPM1, a key downstream target of ILF2. We constructed AAV vectors carrying shNPM1 and subcutaneously injected them into STZ-induced diabetic mice for 4 weeks. The experimental groups included normal control (Non-sh NPM1-ctrl), normal knockdown (non-sh NPM1), diabetic control (DM-sh NPM1-ctrl), and diabetic knockdown (DM-sh NPM1). NPM1 knockdown significantly promoted wound healing. The knockdown groups (non-sh NPM1, DM-sh NPM1) showed faster wound closure compared to control groups (Figure 11a and b). In diabetic mice, the DM-sh NPM1 group exhibited the most rapid healing, with significantly higher wound closure rates on Days 3, 5, and 7 compared to the DM-sh NPM1-ctrl group. Histological analysis (Figure 11c) revealed smaller skin fissures and more intact tissue structure in the DM-sh NPM1 group, indicating reduced tissue damage and cell loss. Western blot analysis (Figure 11d) demonstrated that NPM1 knockdown reduced NPM1 protein expression, decreased p65 phosphorylation levels, and downregulated SASP factors (IL-1β, IL-6, IL-8, MMP1, and MMP3), with more pronounced effects in diabetic mice. Immunofluorescence staining (Figure 11e) further validated that NPM1 knockdown inhibits NF-κB activation.

These results collectively demonstrate that ILF2 plays a key role in diabetic wound healing by negatively regulating NPM1. The ILF2-NPM1 axis modulates the NF-κB signaling pathway and SASP factor expression, thereby alleviating wound repair disorders caused by inflammation-induced senescence.

## Discussion

DFUs are a prevalent complication of diabetes, with a 34% lifetime risk for affected individuals [38]. The global public health burden of DFU is significant, given the need for hospitalization and the potential for amputation. Standard treatments [39], including wound debridement, revascularization, inflammation control, and decompression, are often insufficient. As a result, there is an urgent need for novel therapeutic approaches to improve DFU healing rates.

Wound healing involves four key stages: hemostasis, inflammation, proliferation, and remodeling, which require the coordinated action of various cells and growth factors [4]. Fibroblasts play a pivotal role in this process by synthesizing the extracellular matrix (ECM), promoting angiogenesis, and regulating inflammation to accelerate wound healing. However, in the diabetic microenvironment, HG levels and advanced glycation end products induce oxidative stress, inflammation, mitochondrial dysfunction, and cellular senescence [6, 7]. Senescent fibroblasts exhibit detrimental phenotypes, including secretion of SASP factors, impaired proliferative capacity, and ECM disorganization, which collectively impede wound healing [13, 30]. Recent studies [17, 40, 41] have highlighted the dysregulation of RBPs as a key factor in delayed wound healing, particularly in inflammation and ECM remodeling. However, the role of RBPs in diabetic wounds, especially within fibroblasts, remains poorly understood. In our study, analysis of scRNA-seq data from healing and non-healing DFU tissues (GSE165816) revealed significant transcriptional differences in fibroblasts. Consistent with previous research [27], we identified ILF2 as a key downregulated RBP in DFU by integrating datasets GSE199939, GSE165816, and the RBP2GO database. We further found that HG exposure downregulated ILF2 and upregulated the SASP in fibroblasts, which in turn reduced their proliferation and migration. At the molecular level, we demonstrated that the downregulation of ILF2 leads to increased NPM1 expression, which promotes inflammatory senescence in HG-treated fibroblasts, thereby impairing their proliferative and migratory functions. Additionally, we confirmed that ILF2 binds to NPM1 mRNA, destabilizing it and decreasing its expression. Moreover, ILF2 targets NPM1 to regulate p65 phosphorylation, thereby inhibiting the activation of the NF-κB pathway. The results from animal experiments further demonstrate that ILF2 promotes wound healing in both normal and diabetic mice, while inhibiting the expression of SASP factors. In summary, Our findings highlight the potential of ILF2 as a promising therapeutic target.

Although our study confirms ILF2 downregulation in DFU, the specific mechanisms underlying this change remain to be elucidated. Based on existing literature, several potential pathways may contribute to ILF2 downregulation. For instance, transcriptional regulation could be involved, as HG levels may alter the activity of transcription factors (e.g. JAK-STAT3 [42], Sp1 [43]) that bind to the ILF2 promoter, repressing its transcription. Concurrently, post-transcriptional regulation by microRNAs (miRNAs) [44] represents a plausible mechanism，hyperglycemia alters the expression of numerous miRNAs that target RBPs, potentially destabilizing ILF2 mRNA or inhibiting its translation. Additionally, upstream signaling pathways (e.g. PKC pathway [45]) could be activated by HG, leading to the suppression of ILF2 expression through downstream effectors. While these mechanisms are speculative and require further experimental validation, they provide a framework for future studies to explore the regulatory networks governing ILF2 expression in DFU.

mRNA stability regulation plays a crucial role in gene expression and is one of the key mechanisms through which RBPs control various biological functions. In wound healing research, RBPs have garnered increasing attention. Studies have shown that wound healing involves multiple molecular mechanisms, including inflammatory responses, extracellular matrix remodeling, and cell proliferation. RBPs play a crucial role in diabetic wound repair by regulating the expression of genes associated with these processes. For example, TATA-box binding protein-associated factor 15 (TAF15) was found to stabilize the mRNA of nuclear factor erythroid 2-related factor 2 (Nrf2), activating the Nrf2 signaling pathway and promoting DFU wound healing [20]. Additionally, YTHDC1 [21] regulates the stability of sequestosome 1 (SQSTM1) mRNA, enhancing keratinocyte autophagy and facilitating diabetic wound healing. In our study, we discovered that ILF2 inhibits the secretion of SASP factors in fibroblasts, promoting cell proliferation and migration, which contributes to wound healing in diabetes. Similar findings were observed in macrophages, where Jin *et al*. [25] demonstrated that ILF2 suppresses inflammation. Furthermore, our prior studies [46] also support ILF2’s role in promoting proliferation and migration in keratinocytes, further confirming its importance in wound repair. To elucidate ILF2’s function as an RNA-binding protein (RBP), we used the catRAPID database and identified that ILF2 contains an RNA-binding domain, enabling it to interact with mRNA. Subsequently, by integrating catRAPID with RNA-seq data and the starBase (ENCORI) database, we identified NPM1 mRNA as the highest-scoring target. Using RIP and RNA pull-down assays, We confirmed that ILF2 interacts with NPM1 mRNA. RNA stability assays further demonstrated that ILF2 facilitates the degradation of NPM1 transcripts, leading to a marked reduction in NPM1 protein levels. Consistent with our findings, Li *et al*. also reported that ILF2 functions as an RNA-binding protein involved in the regulation of mRNA stability in esophageal cancer [47]. These findings collectively underscore the critical role of ILF2 in regulating mRNA stability and its potential as a therapeutic target for enhancing wound healing in diabetic conditions.

NPM1 [48] is a critical nucleocytoplasmic shuttling protein involved in several essential cellular processes, including metabolism, DNA repair, and apoptosis. Increasing evidence indicates that NPM1 also plays an important role in transcriptional regulation by interacting with various transcription factors. It has been shown to facilitate transcriptional activation in multiple contexts, including through its interactions with Myc-interacting zinc finger protein 1, the androgen receptor, ribosomal gene promoters, c-Myc, and NF-κB [49–52]. Among these, the NF-κB signaling pathway is particularly noteworthy for its central role in inflammation and cellular senescence, acting as a key mediator in chronic inflammatory diseases. Mounting evidence links NPM1 to NF-κB activity in this context. Rao *et al*. [34]. demonstrated that NPM1 directly interacts with the p65 subunit of NF-κB, promoting its nuclear translocation and enhancing transcriptional activity at the promoters of inflammation-related genes such as IL-1β, IL-6, ICAM-1, and E-selectin. Further supporting this, Lin *et al*. [35]. showed that NPM1 is essential for TNF-α- and LPS-induced inflammatory responses in mouse embryonic fibroblasts, facilitating the transcription of key pro-inflammatory genes including IL-8, TNF-α, and IL-6.Most studies on NPM1 dysregulation to date have focused on its role in cancer, including lymphoma, acute myeloid leukemia, breast cancer, and gastric cancer. However, its function in chronic non-healing wounds, such as DFUs, has not yet been reported. In this study, we found that NPM1 was significantly upregulated under ILF2 knockdown conditions, and this upregulation was accompanied by an increase in p65 phosphorylation levels. Elevated NPM1 expression was also observed in wound tissues from both DFU patients and diabetic mice, supporting its potential pathological role in diabetic wound inflammation. To determine whether NPM1 plays a critical role in this context, we silenced NPM1 expression and performed rescue experiments. The results showed that NPM1 knockdown markedly alleviated the impaired fibroblast proliferation and migration induced by ILF2 silencing, significantly suppressed the expression of SASP factors, and reduced p65 phosphorylation. Additionally, co-immunoprecipitation (Co-IP) assays confirmed the interaction between NPM1 and p65 in fibroblasts, and immunofluorescence analysis validated increased nuclear translocation of p65, consistent with previous reports [34, 35]. In summary, our findings identify NPM1 as a critical downstream effector of ILF2. Through modulation of NF-κB signaling, NPM1 promotes inflammation and impairs wound healing under diabetic conditions. In DFU, chronic activation of NF-κB drives the persistent expression of SASP factors, creating a hostile microenvironment that prevents fibroblast proliferation and matrix remodeling [8]. Our study provides compelling evidence linking the ILF2-NPM1 axis directly to this pathway. Mechanistically, we demonstrated that ILF2 deficiency leads to NPM1 accumulation, which physically interacts with the p65 subunit of NF-κB. This interaction facilitates the phosphorylation of p65 and its subsequent nuclear translocation, thereby amplifying the transcription of downstream pro-inflammatory genes. Rescue experiments further confirmed this hierarchical regulation, as silencing NPM1 effectively reversed NF-κB hyperactivation and restored fibroblast function. Collectively, these findings suggest that the ILF2–NPM1–NF-κB axis is not merely correlative but represents a specific regulatory cascade that explains the transition from metabolic stress to chronic inflammation in diabetic fibroblasts.

Despite these insights, several limitations should be noted. First, the detailed mechanism by which ILF2 binding triggers NPM1 mRNA destabilization remains unclear. Second, the specificity of this interaction among other potential ILF2 targets was not fully addressed. Finally, although key findings were validated in primary cells, most mechanistic experiments were conducted using cell lines rather than primary cells. Addressing these limitations in future research will further strengthen the clinical relevance of the ILF2–NPM1 axis.

Our study identifies ILF2 as a critical RNA-binding protein (RBP) downregulated in DFU fibroblasts, where its deficiency drives impaired wound healing through dysregulation of NPM1-mediated NF-κB signaling. Integrated multi-omics analysis of DFU patient tissues and diabetic models revealed that ILF2 loss destabilizes NPM1 mRNA, leading to NPM1 accumulation. Elevated NPM1 promotes p65 phosphorylation and nuclear translocation, hyperactivating NF-κB to induce inflammatory senescence, characterized by SASP factor secretion, and suppressing fibroblast proliferation and migration. Rescue experiments confirmed NPM1 as the key downstream effector, as its knockdown reversed ILF2 deficiency-induced cellular dysfunction and SASP expression. Functionally, ILF2 restoration accelerated wound healing in diabetic mice by suppressing NPM1/NF-κB-driven inflammation. These findings establish the ILF2–NPM1–NF-κB axis as a novel mechanistic pathway in DFU pathogenesis and highlight ILF2 as a promising therapeutic target to resolve chronic inflammation and promote tissue repair in diabetic wounds.

## Conclusions

In summary, this study first identifies the downregulation of ILF2 in fibroblasts from DFU patients through integrated multi-omics analysis. We further demonstrate that ILF2 regulates NPM1 expression by modulating the stability of NPM1 mRNA, thereby attenuating NF-κB signaling and the associated SASP. These findings highlight the ILF2–NPM1–NF-κB axis as a promising therapeutic target. Potential intervention strategies, such as restoring ILF2 expression or inhibiting NPM1 activity, could offer effective approaches to suppress SASP production and promote wound healing in diabetic patients.

## Supplementary Material

Suppplementary_tkag021

## Data Availability

Data will be made available on request.
