# Supplementary material for: Mechanism of ribonucleic acid-binding protein ILF2 in promoting diabetic foot ulcer wound healing via regulating the nucleophosmin 1/NF-κB axis
Source: Burns Trauma. 2026 Mar 17;14:tkag021. doi: 10.1093/burnst/tkag021 (PMC13313516; doi:10.1093/burnst/tkag021)
Supplement: Suppplementary_tkag021 [file suppplementary_tkag021.docx]

| 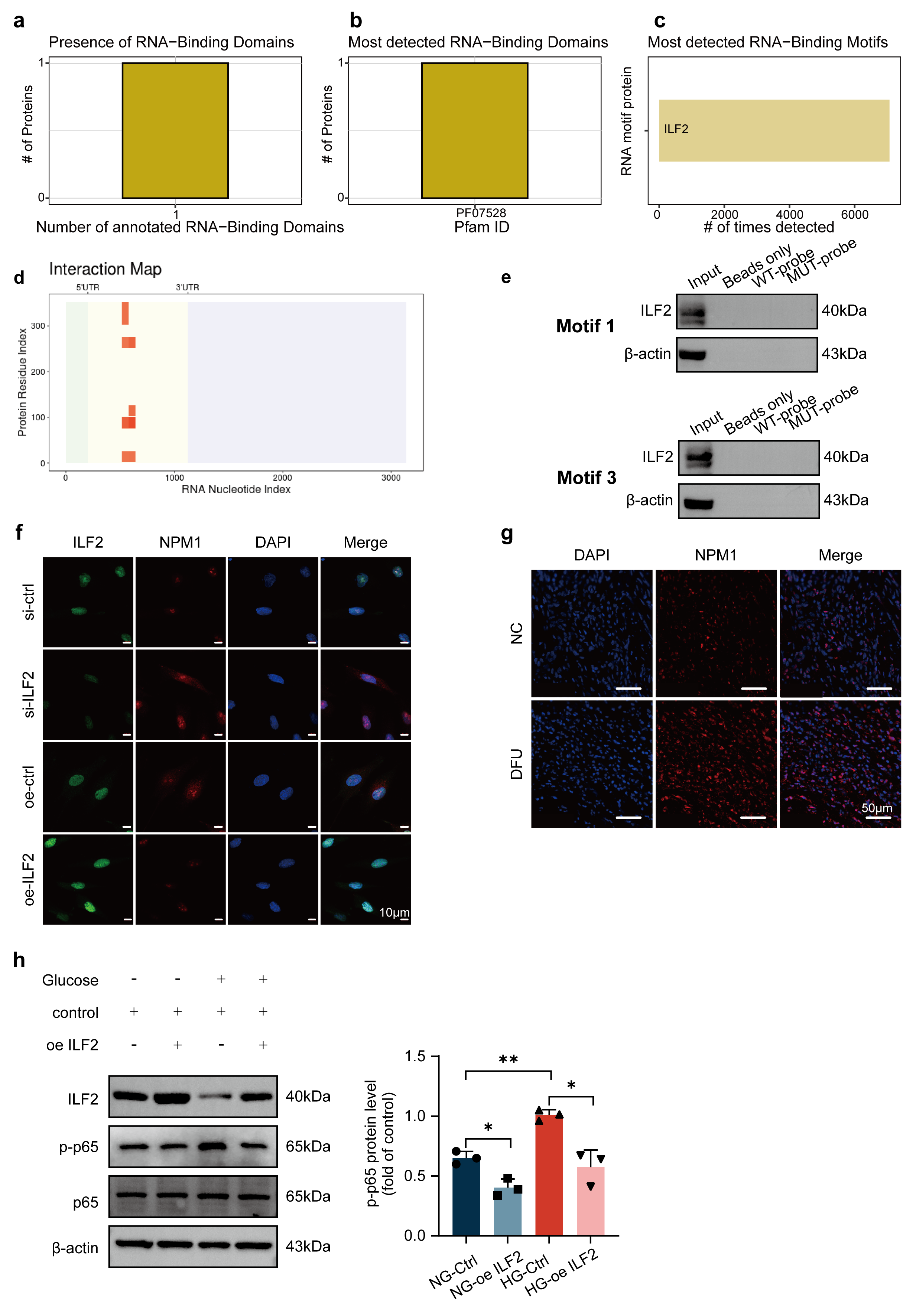 |
| --- |
| **Fig s1**. (a) Presence of annotated RNA-binding domains. (b) Most frequently detected RNA-binding domains (Pfam ID). (c)Most detected RNA-binding motifs. (d) Predicted interaction map between ILF2 and NPM1 mRNA generated by catRAPID, showing putative binding regions along the NPM1 transcript (5′UTR–3′UTR) and ILF2 residues. (e) Western blot analysis of RNA pulldown showing that Motif1 and Motif3 NPM1 mRNA probes could not effectively pull down ILF2 protein. (f) Immunofluorescence staining showing colocalization of ILF2 and NPM1 in fibroblasts (scale bar, 20 μm). (g) Immunofluorescence staining of NPM1 (red) in wound tissues from NC and DFU patients; nuclei were stained with DAPI (blue) (scale bar, 50 μm). (h) Western blot analysis of p-p65 in fibroblasts under NG and HG conditions after ILF2 overexpression.*p < 0.05; **p < 0.01; ***p< 0.001.  Abbreviations: ILF2, interleukin enhancer binding factor 2; NPM1, nucleophosmin 1; UTR, untranslated region; NC, normal control; DFU, diabetic foot ulcer; NG, normal glucose; HG, high glucose; p-p65, phosphorylated p65; DAPI, 4’,6-diamidino-2-phenylindole. |

| 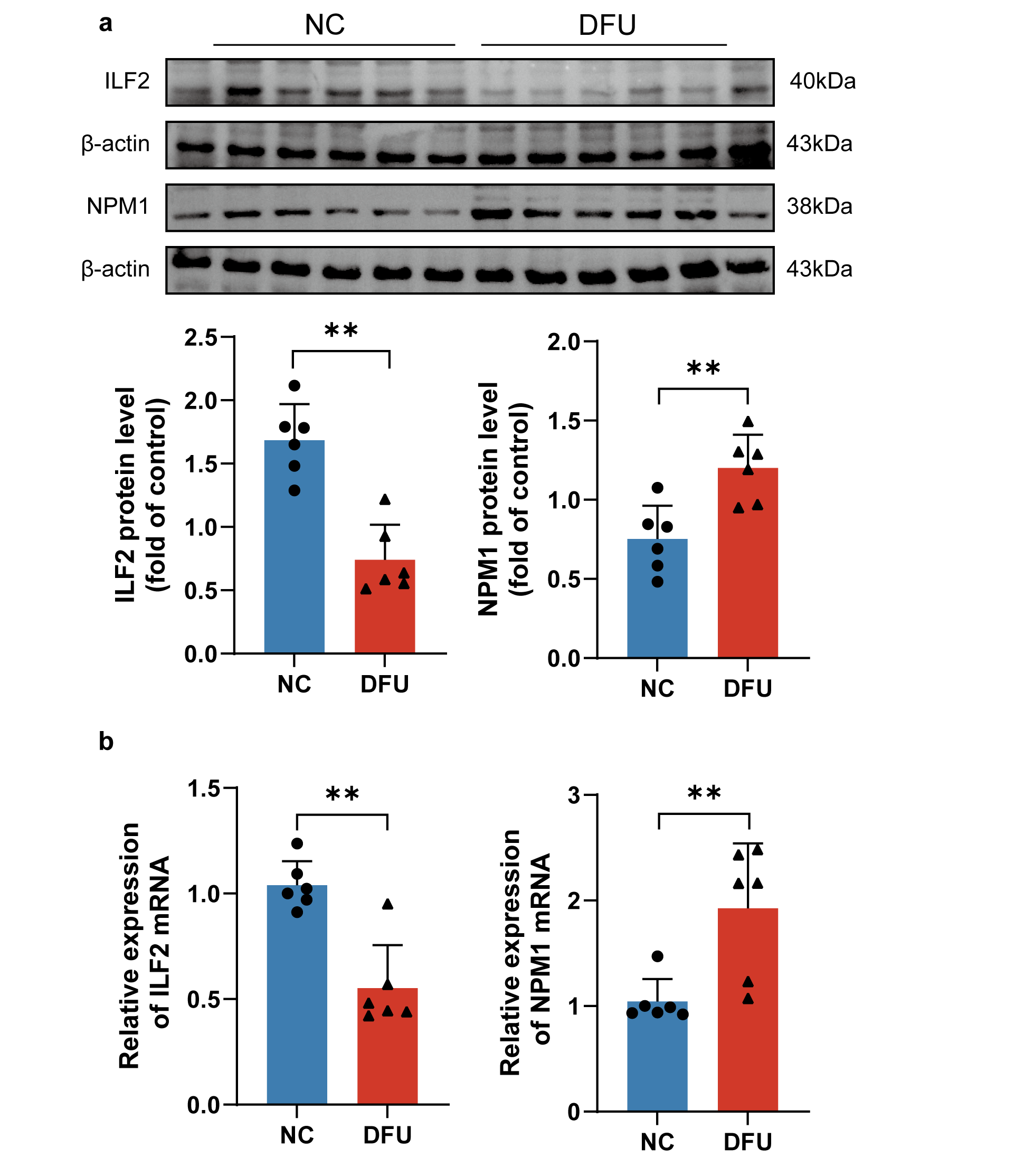 |
| --- |
| **Fig s2. Validation of ILF2 and NPM1 expression in an additional 6 pairs of patient tissues.** (a) Western blot analysis of ILF2 and NPM1 expression in wound tissues from non-diabetic chronic lower limb ulcer (NC) and diabetic foot ulcer (DFU) patients. (b) qPCR analysis of ILF2 and NPM1 expression in wound tissues from patients with non-diabetic chronic lower limb ulcer (NC) and diabetic foot ulcer (DFU). *p < 0.05; **p < 0.01; ***p< 0.001.  Abbreviations: NC, non-diabetic chronic lower limb ulcer; DFU, diabetic foot ulcer; qPCR, quantitative polymerase chain reaction. |

| 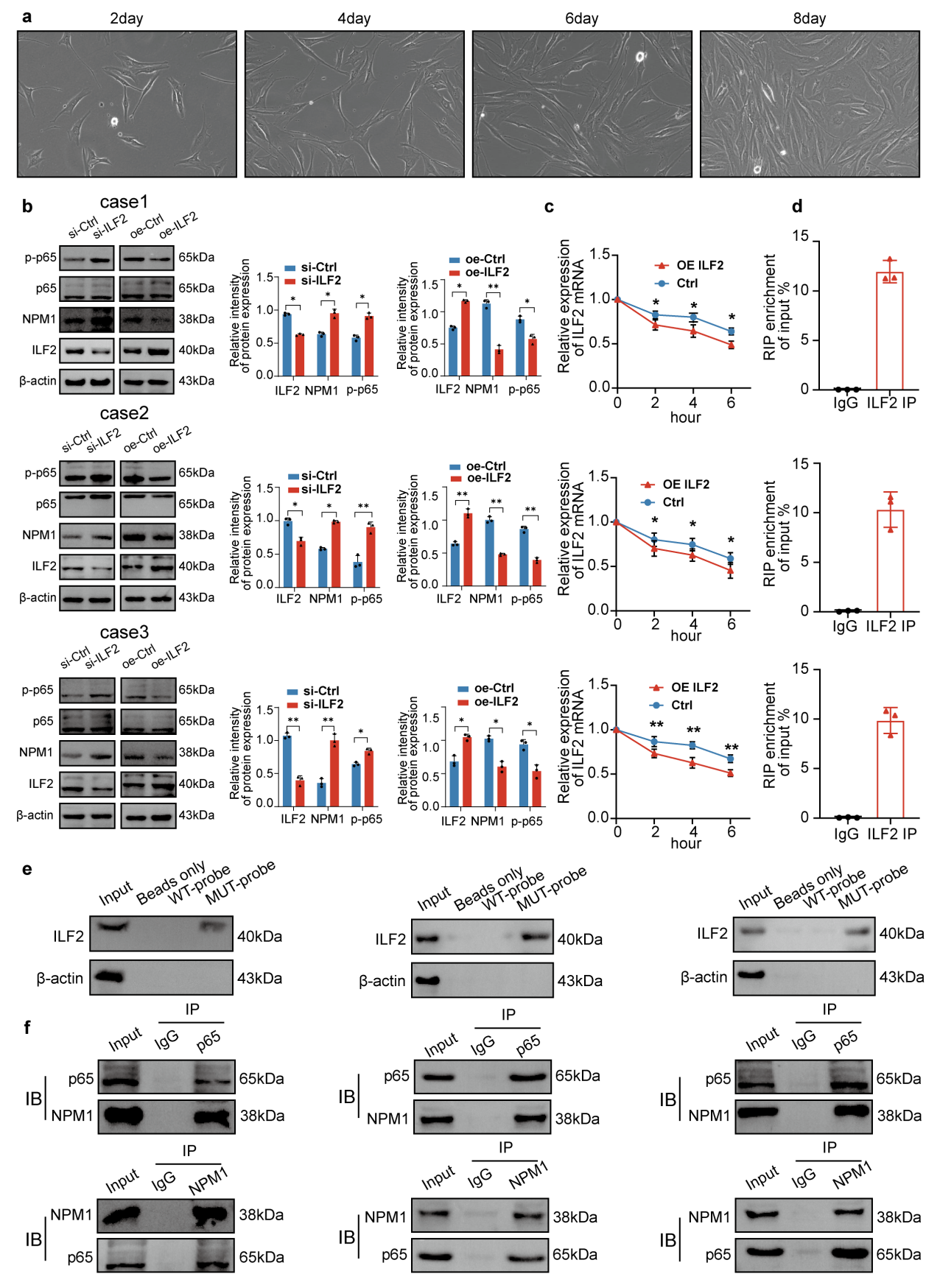 |
| --- |
| **Fig s3. ILF2 regulates NPM1 expression and NF-κB activation by binding to NPM1 mRNA: supplementary verification of the key mechanism in primary skin fibroblasts from wounds of three DFU patients.** (a) Morphological changes in primary skin fibroblasts at different culture time points (Days 2, 4, 6, and 8). (b) Western blot analysis of NPM1 and NF-κB (p65) protein expression levels following ILF2 overexpression (oeILF2) or knockdown (siILF2).(c) mRNA stability assay of NPM1 following ILF2 overexpression in fibroblasts, assessed after actinomycin D treatment. (d) RNA immunoprecipitation (RIP) assay of ILF2 protein with NPM1 mRNA. (e) Western blot analysis showing that the NPM1 motif 2 mRNA probe pulled down ILF2 protein, verifying their direct interaction. (f) Co-immunoprecipitation showing the interaction between NPM1 and p65 in the indicated primary cells. *p < 0.05; **p < 0.01; ***p< 0.001.  Abbreviations: DFU, diabetic foot ulcer; RIP, RNA immunoprecipitation;ILF2,Interleukin enhancer-binding factor 2 ; NPM1, nucleophosmin 1;p65, NF-κB p65; p-p65, phospho-p65. |

| **Table S1.Baseline characteristics of the study participants.** | | | | | | | | |
| --- | --- | --- | --- | --- | --- | --- | --- | --- |
| **Donor** | **Group** | **Gender** | **Age** | **HbA1c**  **(%)** | **BMI** | **Ulcer Etiology** | **Etiology** | **Ulcer Duration**  **(weeks)** |
| 1 | NC | F | 51 | - | 24.46 | Traumatic ulcers | contusion | 4 |
| 2 | NC | M | 35 | - | 25.83 | Traumatic ulcers | laceration | 5 |
| 3 | NC | M | 59 | - | 26.04 | Traumatic ulcers | avulsion injury | 5 |
| 4 | NC | M | 52 | - | 26.93 | Venous ulcers | varicose veins | 6 |
| 5 | NC | F | 53 | - | 27.29 | Venous ulcers | varicose veins | 4 |
| 6 | NC | F | 70 | - | 22.49 | Infectious ulcers | Bacterial infection | 4 |
| 7 | NC | M | 68 | - | 18.37 | Venous ulcers | varicose veins | 7 |
| 8 | NC | M | 62 | - | 24.11 | Venous ulcers | varicose veins | 6 |
| 1 | DFU | M | 72 | 8.1 | 19.94 | - | - | 8 |
| 2 | DFU | M | 50 | 8.6 | 19.03 | - | - | 7 |
| 3 | DFU | F | 76 | 9.6 | 21.6 | - | - | 38 |
| 4 | DFU | M | 57 | 10.3 | 22.6 | - | - | 13 |
| 5 | DFU | M | 51 | 11.5 | 23.66 | - | - | 12 |
| 6 | DFU | M | 54 | 9.7 | 21.97 | - | - | 7 |
| 7 | DFU | F | 60 | 8.7 | 17.53 | - | - | 9 |
| 8 | DFU | M | 51 | 14.5 | 27.75 | - | - | 17 |

Abbreviations:DFU, diabetic foot ulcer. NC,non-diabetic chronic lower limb ulcer.M, male. F, female. HbA1c, Hemoglobin A1C.

| **Table S2.Sequences of ILF2 knockdown constructs** | |
| --- | --- |
| **Gene name** | **Sequences** |
| h-ILF2-si | Forward (5’-3’):CUGGAUCCUUGACCUACUA |
|  | Reverse (5’-3’):UAGUAGGUCAAGGAUCCAG |
| h-NPM1-162 | Forward (5’-3’):GGAUGAGUUGCACAUUGUU |
|  | Reverse (5’-3’):AACAAUGUGCAACUCAUCC |

| **Table S3.The sequences of primer pairs used in RT-qPCR.** | |
| --- | --- |
| **Gene name** | **Sequences** |
| Human ILF2 | Forward (5’-3’): GGGGAACAAAGTCGTGGAAAG |
|  | Reverse (5’-3’): CCAGTTTCGTTGGTCAGCA |
| Human β-actin | Forward (5’-3’): GTGGCCGAGGACTTTGATTG |
|  | Reverse (5’-3’): CCTGTAACAACGCATCTCATATT |
| Human NPM1 | Forward (5’-3’): GGAGGTGGTAGCAAGGTTCC |
|  | Reverse (5’-3’): TTCACTGGCGCTTTTTCTTCA |
| Mouse β-actin | Forward (5’-3’): GGCTGTATTCCCCTCCATCG |
|  | Reverse (5’-3’): CCAGTTGGTAACAATGCCATGT |
| Mouse ILF2 | Forward (5’-3’): GCCCTGAATGTGGCTTACAG |
|  | Reverse (5’-3’): GTGTGTACCCTGAAGTTGCC |
| Mouse NPM1 | Forward (5’-3’): CGGTTGAAGTGTGGTTCAGG |
|  | Reverse (5’-3’): GAGCAGATCGCTTTCCAGAC |

| **Table S4 Antibody information** | | | |
| --- | --- | --- | --- |
| Antibody Type | Target Antigen | Catalog No. | Supplier |
| Primary | β-actin | 2D4H5 | Proteintech |
| Primary | ILF2 | 14714-1-AP | Proteintech |
| Primary | NPM1 | 4F12A3 | Proteintech |
| Primary | IL1β | 26048-1-AP | Proteintech |
| Primary | IL6 | 240670F10 | Proteintech |
| Primary | IL8 | 27095-1-AP | Proteintech |
| Primary | MMP1 | 10371-2-AP | Proteintech |
| Primary | MMP3 | 17873-1-AP | Proteintech |
| Primary | p65 | 4C7 | Proteintech |
| Primary | p65 (Ser536) | 240777D9 | Proteintech |
| Primary | p65 | ET1603-12 | HUABIO |
| Primary | p65 (S536) | HA723223 | HUABIO |

| **Table S5. Comparison of clinical characteristics between DFU-healers and DFU-non-healers.** | | |
| --- | --- | --- |
|  | DFU-Healers | DFU-Non-Healers |
| Number (males) | 7 (2) | 4 (2) |
| Age (years) | 58 ± 18 | 50 ± 11 |
| Diabetes duration (years) | 13 ± 6 | 19 ± 22 |
| BMI (kg/m2) | 35.6 ± 6.08 | 43.1 ± 19.5 |
| HbA1c * (%) | 9.7 ± 3.6 | 7.8 ± 1.1 |
| Creatinine (mg/dL) | 1.1 ± 0.5 | 1.9 ± 1.4 |
| Blood Urea Nitrogen (mg/dL) | 22 ± 14 | 34 ± 23 |
| Cholesterol, Total (mg/dL) | 168 ± 64 | 137 ± 18 |
| Triglycerides (mg/dL) | 123 ± 72 | 272 ± 167 |
| LDL Cholesterol (mg/dL) | 76 ± 52 | 64 ± 10 |
| Wound Surface Area (cm²) | 1.0 (0.2 : 3.8) | 1.2 (0.3 : 22.8) |

Abbreviations: DFU, diabetic foot ulcer.BMI, body mass index; HbA1c, hemoglobin A1c; LDL, low-density lipoprotein.HbA1c, Hemoglobin A1C.

| **Table S6.Major components of the SASP** | | | | | |
| --- | --- | --- | --- | --- | --- |
| category | Group | SASP factors | Senescence-related functions | Senescence inducers of the factors | Upstream regulators |
| Proteins | Interleukins,  cytokines | IL-1α, IL-1β | Activators of other interleukins and of NF-κB, p38 MAPK and mTOR signalling | RS, TIS, OIS, IIS, ageing stressa | NF-κB, p38 MAPK, mTOR |
|  |  | IL-6, IL-8 | Induce autocrine and paracrine senescence; promote immune responses, tumorigenesis and somatic cell reprogramming |  | IL-1α, NF-κB, p38 MAPK, mTOR, cGAS–STING |
|  |  | IL-11 | Associated with ageing and tissue fibrosis | IIS, ageing stress | Sirtuin 1 |
|  |  | IL-33 | Promotes hepatocellular carcinoma progression | Obesity | IL-1β |
|  |  | BAFF | Modulates the immunity by regulating IL-6, NF-κB and p53 | TIS, OIS | IRF1 |
|  | Chemokines | CCL2 | A chemoattractant of monocytes and macrophages; involved in inflammation and tumorigenesis | RS, TIS, OIS, ageing stress | NF-κB, p38 MAPK, mTOR, IL-1α |
|  |  | CCL5 | Promotes tumorigenesis and involved in reproductive ageing | RS, TIS, OIS, ageing stress | NF-κB, p38 MAPK, mTOR |
|  |  | CXCL1, CXCL2, CXCL3 | Promote tumorigenesis, liver regeneration and inflammation | RS, TIS, OIS, ageing stress | NF-κB, p38 MAPK, mTOR, CXCR2, p53 |
|  |  | CXCL5 | Secreted by aged oocytes and is detrimental for in vitro fertilization of pre-implantation embryos | Ageing stress | NF-κB, CXCR2 |
|  |  | CXCL10 | Secreted by senescent hepatocytes to enhance the activities of NK cells | ROS | CXCR3 |
|  |  | CXCL11 | Secreted by senescent endothelial cells; promotes the aggressiveness of breast cancer | TIS | NF-κB, p38 MAPK, mTOR |
|  |  | CXCL14 | In p21-driven SASP, promotes immunosurveillance by recruiting macrophages | TIS, RS, OIS | p21–Rb, STAT–SMAD |
|  | Growth factors | TGFβ | Promotes paracrine senescence | OIS | Notch |
|  |  | GDF15 | Promotes cancer progression, osteoarthritis and is associated with ageing | TIS, ageing stress | p53, NF-κB |
|  |  | HGF | Promotes liver cancer progression | TIS, OIS | NF-κB |
|  | Proteases | MMP1 | Associated with tissue damage and skin ageing | TIS, OIS,UVB | NF-κB,DDR |
|  |  | MMP3 |  |  |  |
|  | Others | IGFBP3 | In p53-driven and p21-driven SASP, promotes immunosurveillance | TIS, RS, OIS | p53, p21–Rb |
|  |  | LIF | In p53-associated SASP, promotes immunosurveillance | TIS | p53 |
|  |  | ISG15 | Downstream of interferon signalling and enriched in p53-associated SASP | TIS | Type I interferon, p53 |
| Type I interferon, p53 | | Prostaglandins | Promotes tissue repair | TIS, RS | COX2 |
|  |  | Leukotrienes | Involved in tissue fibrosis | TIS | ALOX5 |
| Small extracellular vesicles | | Exosomes | Promote tumorigenesis and paracrine senescence | OIS | EPHA2, IFITM3 |
| Non-coding nucleic acids | | MicroRNAs | Mediate the communication between senescent fibroblasts and keratinocytes | ROS | Small extracellular vesicles |
|  |  | Cytoplasmic chromatin DNA fragments | Elicit DDR in neighbouring cells | RS, OIS | DDR, DNASE2, small extracellular vesicles |
| Abbreviations:ALOX5, polyunsaturated fatty acid 5-lipoxygenase; BAFF, B cell-activating factor; CCL, C-C motif ligand; cGAS–STING, cyclic GMP–AMP synthase–stimulator of interferon genes; CXCL, C-X-C motif ligand; CXCR3, C-X-C motif chemokine receptor 3; DDR, DNA damage response; DNASE2, DNA-degrading enzyme deoxyribonuclease 2; EPHA2, ephrin type A receptor 2; GDF15, growth differentiation factor 15; HGF, hepatocyte growth factor; IFITM3, interferon-induced transmembrane protein 3; IGFBP3, insulin-like growth factor-binding protein 3; IIS, immunologically induced senescence; MMP, matrix metalloproteinase; NK, natural killer; OIS, oncogene-induced senescence; ROS, reactive oxygen species; RS, replicative senescence; SASP, senescence associated secretory phenotype; TGFβ, transforming growth factor-β; TIS, therapy-induced senescence; UVB, ultraviolet B. aAgeing stress comes from cellular and DNA damage accumulated during the natural ageing process. | | | | | |
